# A signed network perspective on the government formation process in parliamentary democracies

**DOI:** 10.1038/s41598-021-84147-3

**Published:** 2021-03-04

**Authors:** Angela Fontan, Claudio Altafini

**Affiliations:** Division of Automatic Control, Department of Electrical Engineering, Linköping University, 58183 Linköping, Sweden

**Keywords:** Complex networks, Applied physics

## Abstract

In parliamentary democracies, government negotiations talks following a general election can sometimes be a long and laborious process. In order to explain this phenomenon, in this paper we use structural balance theory to represent a multiparty parliament as a signed network, with edge signs representing alliances and rivalries among parties. We show that the notion of frustration, which quantifies the amount of “disorder” encoded in the signed graph, correlates very well with the duration of the government negotiation talks. For the 29 European countries considered in this study, the average correlation between frustration and government negotiation talks ranges between 0.42 and 0.69, depending on what information is included in the edges of the signed network. Dynamical models of collective decision-making over signed networks with varying frustration are proposed to explain this correlation.

## Introduction

In a country adopting a multiparty parliamentary system, the process of forming a government after a general election can be quick, or can sometimes be impervious and characterized by lengthy negotiations. Indeed, especially in the last decade, many European countries have experienced unusually long cabinet bargaining phases, for instance Belgium in 2010 and 2019, Bosnia Herzegovina in 2010 and 2018, Croatia in 2015, Czech Republic in 2006, Germany in 2017, Ireland in 2020, Italy in 2018, North Macedonia in 2016, Netherlands in 2017, Norway in 2017, Spain in 2016 (and 2015), Sweden in 2018 and UK in 2018. Typically, long delays appear when a parliament is fragmented and a clear majority is missing: parties have to enter multiple bargainings with each other in order to form a coalition able to win a parliamentary confidence vote.

To model this government formation process, descriptive statistics and game-theoretical models of bargaining have often been used in the literature, see^1–9^ and references therein. These models use the data to fit coefficients expressing the relative importance of certain factors, like number of parties and Members of Parliament (MP), seats per party, ideological polarization, etc., in predicting the cabinet formation times.

One of the important determinants of a government formation timeline is the fragmentation of a legislature, measured for instance in terms of the Laakso–Taagepera effective number of parties^10^ or of the related fractionalization index^11^. This and other factors, such as the ideological diversity of a legislature, the idea of minimum winning coalition^2^, etc., can be used in descriptive statistical models to evaluate how different features influence the government formation process^4,6,8^. For instance, the Cox hazard model of^4^ groups factors into “bargaining complexity” (n. of parties and seats per party, ideological polarization, requirements for the investiture, type of parliamentarism—“negative” or “positive”, see^1^) and “uncertainty” of the political landscape (previous history of collaborations or defeats among the parties, whether the government is a post-electoral or an inter-electoral one, etc.) and uses empirical data to estimate the importance of the different terms.

The literature on bargaining models applied to coalition formation is vast, see for instance^7,9,12–16^ (see also^17^ for an overview). A typical bargaining procedure is organized in two stages: first a player (often the formateur) proposes a coalition of parties and a certain allocation of government portfolios among them, then the members of the candidate coalition can decide to either accept or refuse the proposal. A government is formed only if all the candidate parties agree on the proposal, otherwise the bargaining process continues until a government is formed. This model is used for instance in^18^ to perform an empirical analysis in 11 parliamentary democracies, using electoral data from 1945 to 1997. More complex bargaining procedures are discussed e.g. in^9^.

Departing from the aforementioned literature, this paper aims to propose a novel approach to model the process of government formation, rooted in the social network theory of multiagent systems with antagonistic interactions^19,20^. In particular we represent antagonism among parties as a signed graph, and consider the process of government formation as a collective decision-making over such signed network.

More specifically, our approach relies on the concept of structural balance^21–25^ and of graph frustration, intended as distance from a structurally balanced situation. The idea of structural balance is well-known in social network theory^19,20^, and easily exemplified by notions like “the enemy of my enemy is my friend”: when a signed graph can be partitioned into two subgroups such that all nodes in each subgroup are connected by positive (i.e., “friendly”) edges and all edges across the two subgroups are instead negative (i.e., “unfriendly”) then the graph is structurally balanced and shows no frustration. A two-party parliament (e.g., Malta, if we restrict to European nations) is structurally balanced: all cycles on the signed graph are positive (i.e., have an even number of negative edges). For a structurally balanced parliament, the government formation process is typically straightforward: the party winning the elections is in charge of forming a cabinet. Signed graphs that are not structurally balanced have instead some amount of negative cycles (i.e., cycles with an odd number of negative edges). The notion of *frustration* can be used to quantify the distance to structural balance induced by these negative cycles^25^. This notion, adopted from the statistical physics literature, is expressed as the energy in the ground state of an “Ising spin glass”^25–27^ and quantifies the amount of “disorder” contained in the system.

Our hypothesis is that in parliamentary networks this disorder strongly influences the process of government formation. Namely, the higher the amount of frustration, the longer the government negotiation phase is expected to be. Indeed if there is no clear winner after the elections (i.e., no political party or alliance managed to secure a majority in the parliament) then the parties have to negotiate and overcome their ideological differences in order to form a coalition cabinet backed by the majority in the parliament. The data we collected from 29 European nations over the last 30–40 years show that indeed the frustration of the parliamentary networks correlates well with the duration of the government negotiations talks. This correlation is due to a large extent to the fragmentation of a legislature. In fact, interestingly enough, we show that in the simplest scenario we consider (referred to as scenario **I** below) frustration combines together the notions of fractionalization index and minimum winning coalition.

The correlation between frustration and duration of the government negotiations can be explained also at a deeper level, using dynamical models of collective decision-making in multiagent systems^28–30^. Such models represent a decision as the trespassing of a bifurcation threshold, and the corresponding bifurcation parameter has the interpretation of “social commitment” of the agents, i.e., intensity of the interactions among the agents. In presence of signed graphs, it is known that the bifurcation threshold can be pushed to higher values of the bifurcation parameter, and in particular that its value is proportional to the frustration encoded in the signed network^31^. If we consider as decision a confidence vote on a candidate cabinet, and as “social commitment” the intensity of the government negotiation talks (quantified as duration of the bargaining phase), then in a multiagent dynamics perspective the positive correlation we find between frustration and duration of the government negotiation phase is an expected and reasonable property: more “disordered” parliaments require longer negotiations to form a government. By capturing and quantifying this disorder, the frustration of a parliamentary network allows to predict the government formation timing and also, to some extent, the composition of parties that managed to form a successful cabinet coalition.

## Results

### Correlation between frustration and duration of the government negotiation talks

**Figure 1:**
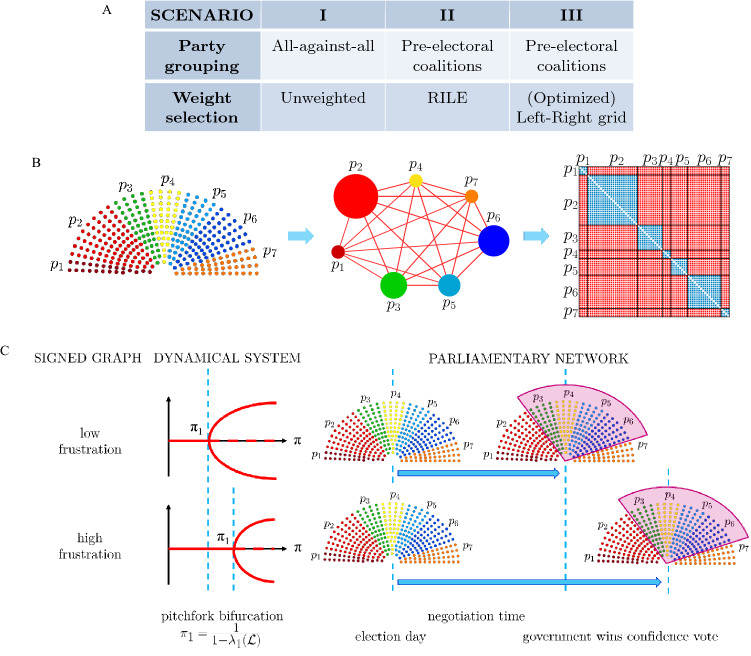
(**A**) The three different scenarios considered in this study. (**B**) Constructing a parliamentary network and the corresponding adjacency matrix in scenario **I** (all parties against all parties and weights equal to $+ 1$, in blue, and $-1$, in red). Each node $p_i$ represents a political party. See Supplementary Fig. 1A for scenario **II** and **III**. (**C**) Process of government formation described as a dynamical model of decision-making over signed networks: the higher is the frustration of the network, the higher is the value of the bifurcation point $\pi _1=\frac{1}{1-\lambda _1(\mathcal {L})}$ ($\lambda _1(\mathcal {L})$ = least eigenvalue of the normalized Laplacian of the signed graph). In turn, the higher is $\pi _1$, the longer is the expected government negotiation time between the parties.

In the 29 European parliamentary democracies or constitutional monarchies considered in this study, see Table 1 (and Supplementary Table 1 for more details), we computed the frustration associated to the parliamentary network resulting after each general election (see “Methods” and “Supplementary Information”). Its value depends on what information is encoded in the weights of the parliamentary network: three different scenarios (denoted **I** to **III**, see Fig. 1A) are considered in this study, from no a-priori information at all on the parties and their relationships, to ideological and electoral coalition information. In particular, in scenario **I** a parliamentary network is built using information on (1): n. of parties of a legislature, and (2): n. of MPs per party. In scenarios **II** and **III** also (3): pre-electoral party coalitions, and (4): ideological polarization of the parties are considered in forming the adjacency matrices of the parliamentary networks. More details on these four factors are provided below, in the “Methods” section (see also Fig. 1B), and in the “Supplementary Information” (see also Supplementary Fig. 1).

**Table 1:** List of countries considered in this study.

Country	System of government	Pre-electoral coalitions	Number of elections	
	(PR or PCM)	✓ = yes or ✗ = no	*N*	(from-to)
Albania	PR	✓	8	(1992–2017)
Andorra	PCM	✓	8	(1993–2019)
Austria	PR	✗	13	(1979–2019)
Belgium	PCM	✓	7	(1995–2019)
Bosnia Herzegovina	PR	✗	8	(1996–2018)
Bulgaria	PR	✗	9	(1991–2017)
Croatia	PR	✓	9	(1992–2020)
Czech Republic	PR	✓	8	(1992–2017)
Denmark	PCM	✗	13	(1981–2019)
Estonia	PR	✗	8	(1992–2019)
Finland	PR	✓	8	(1991–2019)
Germany	PR	✓	8	(1990–2017)
Greece	PR	✗	11	(1990–2019)
Hungary	PR	✓	6	(1990–2010)
Iceland	PR	✗	8	(1995–2017)
Ireland	PR	✓	7	(1992–2020)
Italy	PR	✓	8	(1992–2018)
Latvia	PR	✗	9	(1993–2018)
Luxembourg	PCM	✗	8	(1984–2018)
North Macedonia	PR	✓	10	(1990–2020)
Moldova	PR	✗	8	(1994–2019)
Netherlands	PCM	✗	12	(1981–2017)
Norway	PCM	✓	10	(1981–2017)
Serbia	PR	✗	6	(2007–2020)
Slovakia	PR	✓	9	(1990–2020)
Slovenia	PR	✗	8	(1992–2018)
Spain	PCM	✓	9	(1989–2019)
Sweden	PCM	✓	11	(1982–2018)
United Kingdom	PCM	✓	10	(1983–2019)

For each country the following are shown: the system of government (PR: Parliamentary Republic, PCM: Parliamentary Constitutional Monarchy), the existence of pre-electoral coalitions, the number of general elections considered and their time span.

**Figure 2:**
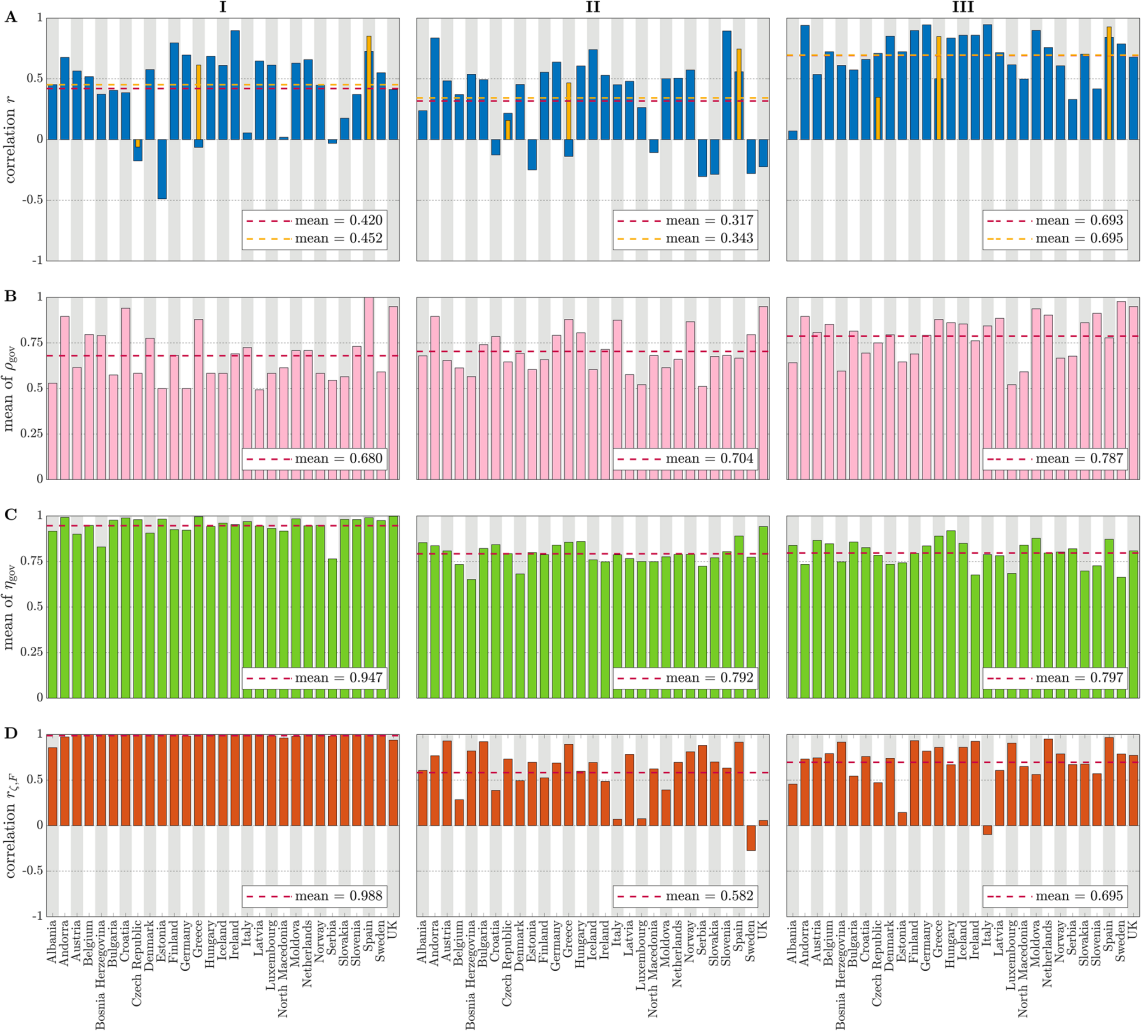
(**A**) Correlation between frustration and duration of the government negotiation talks, quantified in number of days from the general election to the date the government is sworn in or to the date the government negotiations fail, for each country and scenarios **I**, **II** and **III**. Blue bars consider only the cases of success, yellow bars consider both successes and failures. (**B**) Index $\rho _{\text {gov}}$ (overlap between party coalition entering in the government and party majority in the ground state); (**C**) index $\eta _\text {gov}$ (energy gap between ground state and “government state”). (**D**) Correlation between frustration and fractionalization index ($r_{\zeta ,F}$), for each country and scenarios **I**, **II** and **III**. The average values (of correlation, in (**A**,**D**), or of the indexes, in (**B**,**C**)) are reported inside each plot.

Even for the most basic scenario (**I**: the parties are “all-against-all”, which leads to an unweighted fully-connected signed graph with on-diagonal blocks of $+1$ and off-diagonal blocks of $-1$, see Fig. 1B), our calculations show that the frustration of a parliamentary network is indeed a good indicator of the complexity of a post-electoral government formation process: the average correlation (defined by the Pearson correlation coefficient) between the frustration ($\zeta$, computed according to the formula (2), see Supplementary Fig. 2B) and the length of the government negotiation phase (computed as number of days between the general election and the day the new government is sworn in, see Supplementary Fig. 2A) is 0.42, see Fig. 2A. In this scenario, what is modeled is essentially only the fragmentation of a legislature, i.e., n. of parties and n. of MPs per party, and in fact in this case the frustration is nearly identical to the fractionalization index *F* (correlation is 0.99, see Fig. 2D) with a very minor correction needed only when in a country no coalition exists able to achieve exactly 50% $+1$ of the seats. An analytical formula relating these quantities is provided in the “Methods”, see also “Discussion” for more details.

Given the many unmodeled factors constraining and influencing the cabinet bargaining process (see e.g.^32^ Chapter 9 for an overview) we find it remarkable that this level of correlation can be achieved by such simple models, especially since a clear reason behind the lack of correlation exists for several of the countries showing the worst fit (e.g. Czech Republic, Estonia and Greece), see “Discussion”.

More complex adjacency matrices for our parliamentary networks can be formed if we include information such as pre-electoral party coalitions, if available (explicit or implicit, depending on the country, see Table 1), and ideological classification of parties. When for the latter factor we use standard indexes such as *rile* (based on the electoral manifestos of the parties, see^32–34^) then the correlation between network frustration and negotiation days is slightly lower, with an average value of 0.32 (scenario **II** in Fig. 2A). When instead the edge weights are optimized based on a predetermined left-right grid (see Supplementary Fig. 1C and “Supplementary Information” for the details) then the correlation grows up to 0.69 in average (scenario **III** in Fig. 2A). In both scenarios **II** and **III**, frustration and fractionalization index no longer coincide, see Fig. 2D. Because of the weight optimization, scenario **III** cannot be considered parameter-free, unlike scenarios **I** and **II**. To motivate (and validate) the use of this scenario we have performed a “leave-one-out” analysis, where the optimization of the political positions of the parties in the left-right scale is performed only on $N-1$ elections (denoted “training set”, where *N* is the number of elections for each country, see “Methods” for more details) and the remaining election (denoted “validation set”) is used to evaluate how well the model fits the data (in terms of correlation). As Fig. 4 shows, scenario **III** is in general performing well in the leave-one-out analysis. A special case this analysis, when the excluded election is the last one, can be interpreted as the capacity of the model to predict the length of a future negotiation process based only on the frustration of a newly elected parliament. Also in this case the predictive power of the model is in most cases reasonably good, see Supplementary Fig. 10 and the “Discussion” for more details.

**Figure 3:**
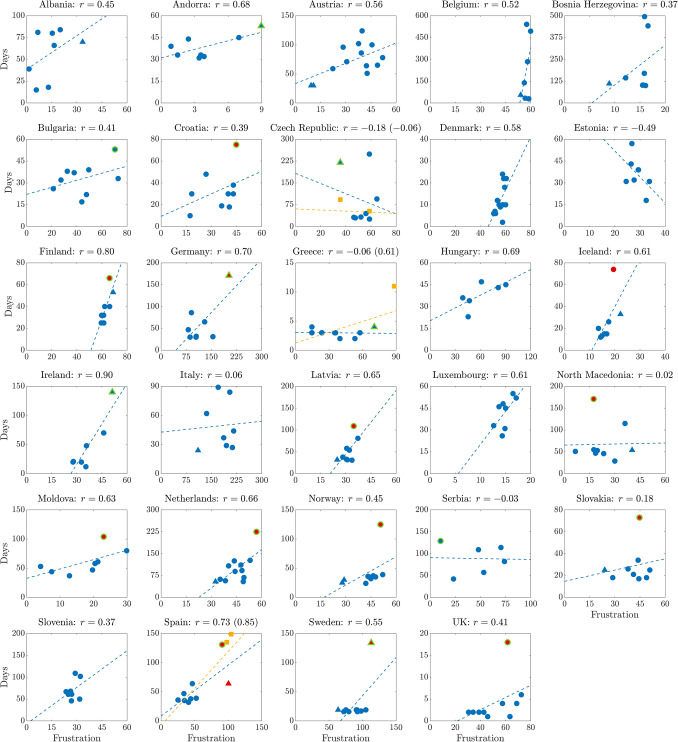
Scenario **I**. Frustration of the parliamentary networks v.s. duration of the government negotiation talks (days) and corresponding linear regression line, for all countries of Table 1. The value of Pearson correlation (*r*) for each country is reported in the plot heading. Legend: blue circles represent points that are neither outliers, nor high leverage nor influential. A red symbol indicates an outlier, a triangle a high leverage point and a symbol with green outline an influential point. Residual analysis, leverage statistic and delete-1 statistics are used to identify outliers, high leverage and influential points, respectively. Yellow square data points indicate elections corresponding to failure of government negotiations resulting in votes of no-confidence (Czech Republic in 2006 and 2017) and new elections (Spain in December 2015 and April 2019, Greece in May 2012). Blue regression lines include only the successful government formations. Including also the failure points we obtain the yellow regression lines. In all three cases, the correlation increases (values are reported between parentheses).

**Figure 4:**
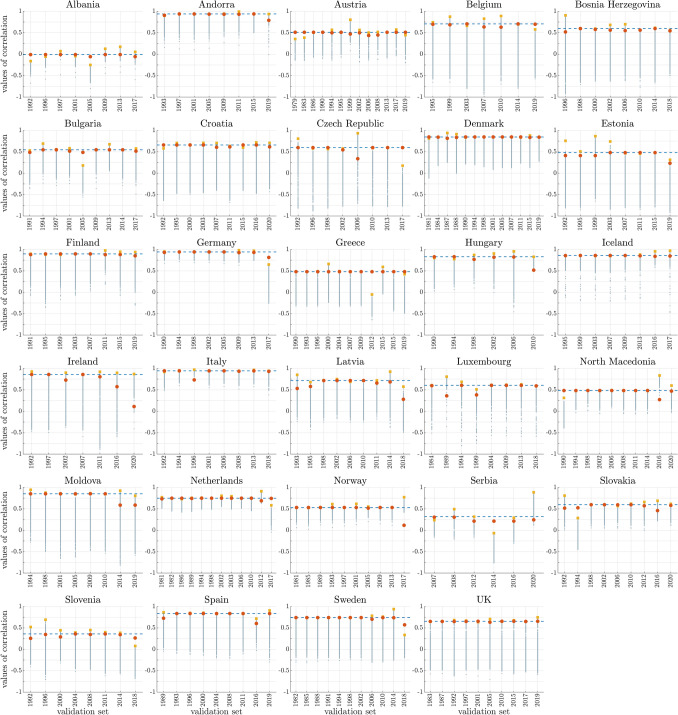
Leave-one-out analysis of the correlation for scenario **III**. A single election is used for the validation set, and the remaining elections (training set) are used to calculate the correlation (frustration v.s. duration of the government negotiation talks) for each choice of political positions in the left-right grid. 1000 sets of values for the left-right political positions of the parties were randomly selected on the preassigned left-right grid. Here the corresponding correlation values for each set are shown (gray dots), together with the overall country maximum value (yellow square) corresponding to the optimal choice for the weights. Red circles represent the values of correlation obtained when the excluded election is considered (the frustration is calculated using the optimal choice for the weights found using the training set). A blue dashed line represents the value of correlation obtained if the weights are tuned so as to maximize the correlation when all the elections are considered (i.e., scenario **III**). The red circles are normally very close to the yellow squares, meaning that the leave-one-out test is normally accurate.

### Prediction of the successful cabinet coalition

The notion of frustration of a parliamentary network can be used also to predict the successful cabinet composition ensuing from the negotiations. If in our signed graphs we assign a “spin” variable (polarized into $\pm 1$ values, corresponding to “spin up” and “spin down”, and here interpretable as yes/no in a confidence vote) to all parties, then it is possible to compute an energy-like function for each spin configuration, as well as for the party coalition that succeeds in forming a post-election government. The frustration then corresponds to the global minimum of this energy functional (i.e., the energy in the ground state, denoted $S_{\text {best}}$). The index $\rho _{\text {gov}}$ described in “Methods” represents the overlap between the party coalition that succeeded in forming a government, $S_{\text {gov}}$, and the majoritarian group of parties in the ground state $S_{\text {best}}$. In our data, $\rho _{\text {gov}}$ varies between a 68% of scenario **I** and a 79% of scenario **III**, see Fig. 2B. It is worth remarking that for several countries (e.g., Czech Republic, Denmark, Norway, Sweden) the overlap is reduced by the fact that minority governments are supported (actively or passively, e.g., through abstentions in confidence votes) by other parties which do not figure explicitly in the cabinet coalition (and hence do not appear in the spin configuration $S_{\text {gov}}$ we use to quantify $\rho _{\text {gov}}$). The lower value of $\rho _{\text {gov}}$ for scenario **I** is also due to the parliamentary network being an unweighted matrix, which leads to many states of near-identical energy, see Supplementary Fig. 3. For weighted adjacency matrices (scenario **II** and **III**) such degenerate cases are much less frequent and in fact $\rho _{\text {gov}}$ increases. Indeed, if we look at the energy gap between ground state and “government state” (i.e., $S_{\text {best}}$ and $S_{\text {gov}}$, see “Methods”), expressed by the index $\eta _{\text {gov}}$ described in the “Methods” section, then it is $\eta _{\text {gov}}> 90\%$ in scenario **I**, even better than for the remaining scenarios ($\eta _{\text {gov}}= 79.2\%$ in scenario **II** and $\eta _{\text {gov}}= 79.7\%$ in scenario **III**, see Fig. 2C), which confirms that even when non-optimal, the successful government coalition is always “energetically” close enough to the ground state coalition $S_{\text {best}}$ identified by our method.

**Figure 5:**
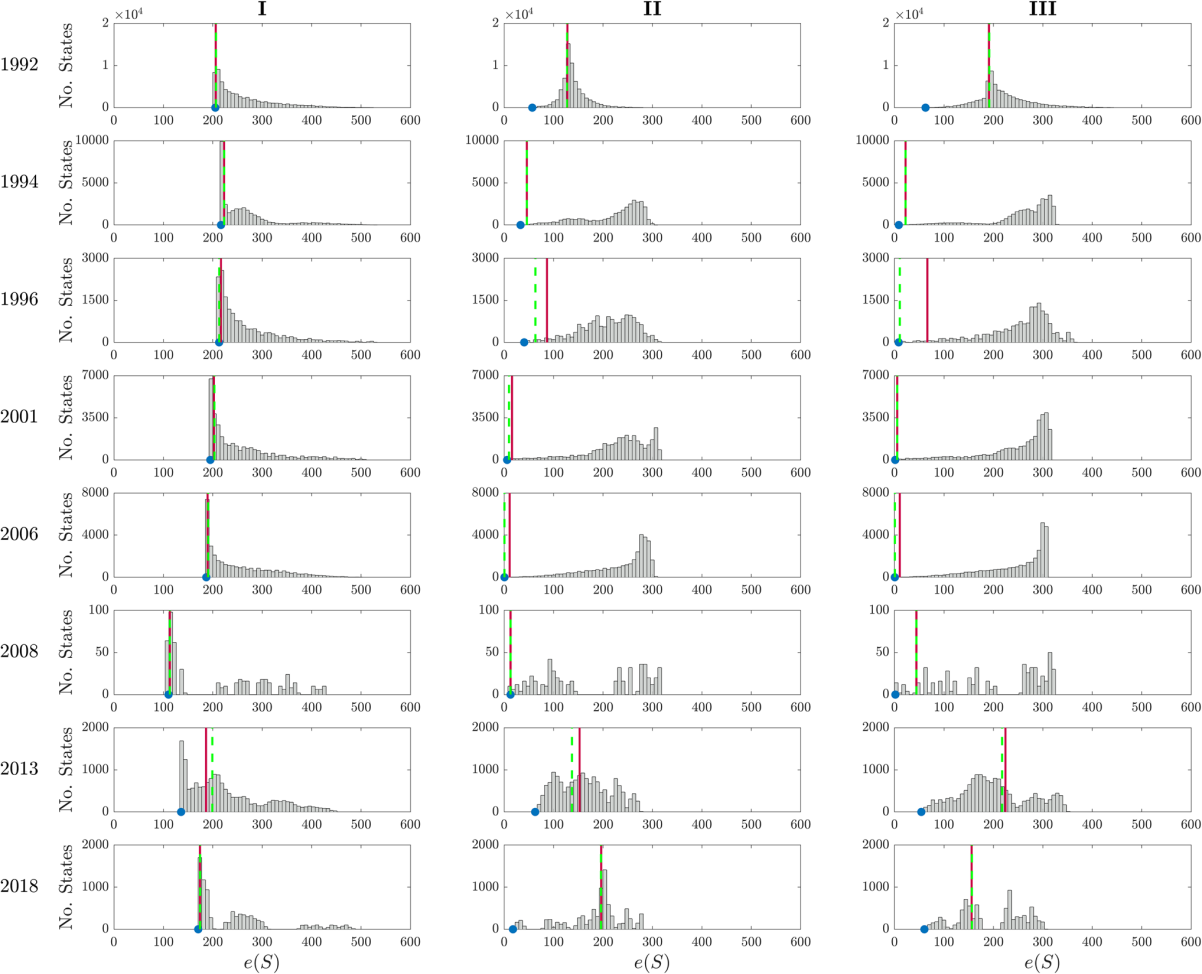
Energy landscape in Italy for each scenario (**I**, **II** and **III**) and election, i.e., values of the energy *e*(*S*) calculated according to Eq. (1) for all the $2^{n_{\text {p}}}$ (${n_{\text {p}}}=$ no. parties in the parliament) choices of $S = \text {diag}\{S_1,\ldots ,S_{n_{\text {p}}}\}$, $S_i = s_i I_{c_i}$, $s_i=\pm 1$. The blue dot indicates the minimum of such energy functionals, i.e., the frustration (2). The red line represents $e(S_{\text {gov}})$, i.e., the value of the energy functional corresponding to the government, while the dashed green line represents $e(S_{\text {gov}+\text {supp}})$, i.e., the value of the energy functional corresponding to the government plus the other parties providing support in parliament without formally entering into the government coalition (see “Methods”).

### Interpretation as collective decision dynamics

The high correlation between frustration and government negotiation days admits an interpretation in terms of dynamical models of collective decision-making, see “Methods” and “Supplementary Information”. These models, used for instance in^30,31^, are inspired by the behavior of animal groups^28,29,35^ and in our case associate a state variable to each MP, variable that represents the decision in a confidence vote. In order for a government to win a confidence vote, an attractor corresponding to a majority of positive decisions must be present. The government formation process is represented in this model by means of a bifurcation occurring for a certain value of a scalar parameter $\pi$ which we can refer to as strength of the “social commitment” among the parties, here intended as a proxy for the duration of the cabinet negotiations process. Increasing $\pi$, the system passes from having the origin as the only globally asymptotically stable equilibrium to a situation in which two extra nonzero states of decision, i.e., two locally asymptotically stable equilibria, are present, while the origin becomes unstable, see Fig. 1C. In the model, the origin represents a state of “no decision” (the MPs do not take any side), while the two stable equilibria appearing after the crossing of the bifurcation point correspond to success and failure of a confidence vote. Since the bifurcation is of pitchfork type and the network is symmetric, these two equilibria are identical up to a change of sign, and they represent a partition of the parliament into two factions, the majoritarian one being the winner of the confidence vote. The bifurcation point is a function of the least eigenvalue of the normalized Laplacian of the network, $\lambda _1 (\mathcal {L})$, see Eq. (8). The latter is called in the signed graph literature the *algebraic conflict*^24^, and it is well-known to be closely related to the frustration^24,31,36^, see Supplementary Table 2 for an analysis on our data. Similarly, the signature of the corresponding eigenvector overlaps substantially with the signature of the ground state spin configuration $S_{\text {best}}$, see Supplementary Table 2. Combining the proportionality between $\lambda _1(\mathcal {L})$ and frustration with Eq. (8), we get a relationship between the value of $\pi$ at the bifurcation point, $\pi _1$, and the frustration of the network. Interpreted in the context of a government formation process, this relationship states that the duration of the government negotiation talks (a proxy of $\pi _1$) is directly proportional to the frustration of the parliamentary network, see “Methods”. When the frustration increases, the model predicts that the bifurcation threshold increases as well (see^31^ and also Supplementary Fig. 4), meaning that a higher commitment will be required from the agents in order to escape a state of no decision and to reach a collective nontrivial equilibrium point. This translates in our model into longer negotiation times for the government formation phase. The concept is illustrated in Fig. 1C.

**Figure 6:**
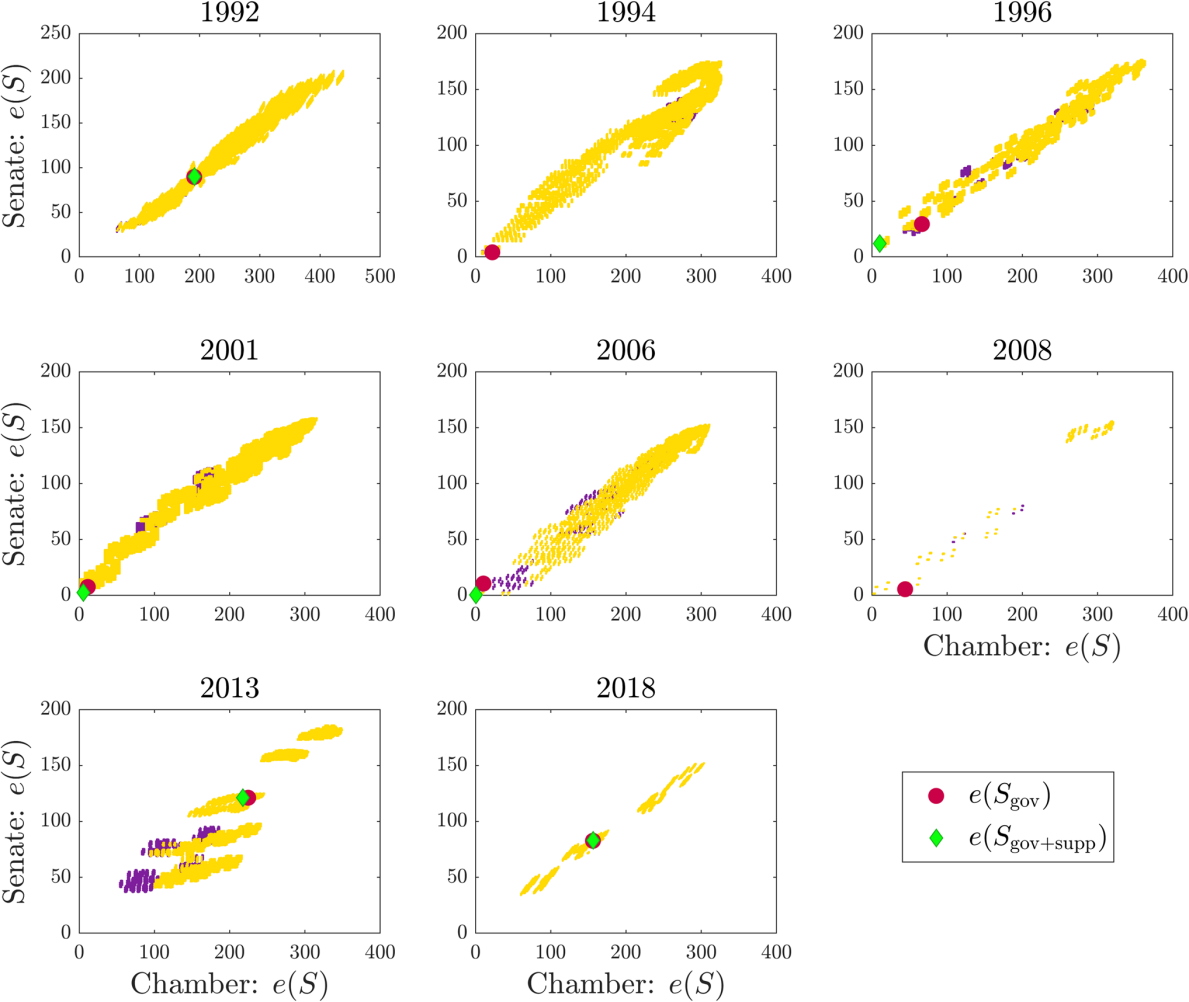
Comparison of the energy landscapes in the Chamber of Deputies and Senate of the Republic in Italy for scenario **III**. The red dot indicates the value of the energy functional corresponding to the government configuration $S_{\text {gov}}$, while the green diamond indicates the value of the energy functional corresponding to the party configuration $S_{\text {gov}+\text {supp}}$, which includes also the parties providing external support to the government in the parliament (see “Methods”). The values of the energy functional corresponding to majority configurations (i.e., party coalitions holding majority of seats in both chambers of the parliament) are highlighted with a yellow color, while a purple color indicates party coalitions which have the majority of seats in only one chamber (see “Supplementary Information” for details).

**Figure 7:**
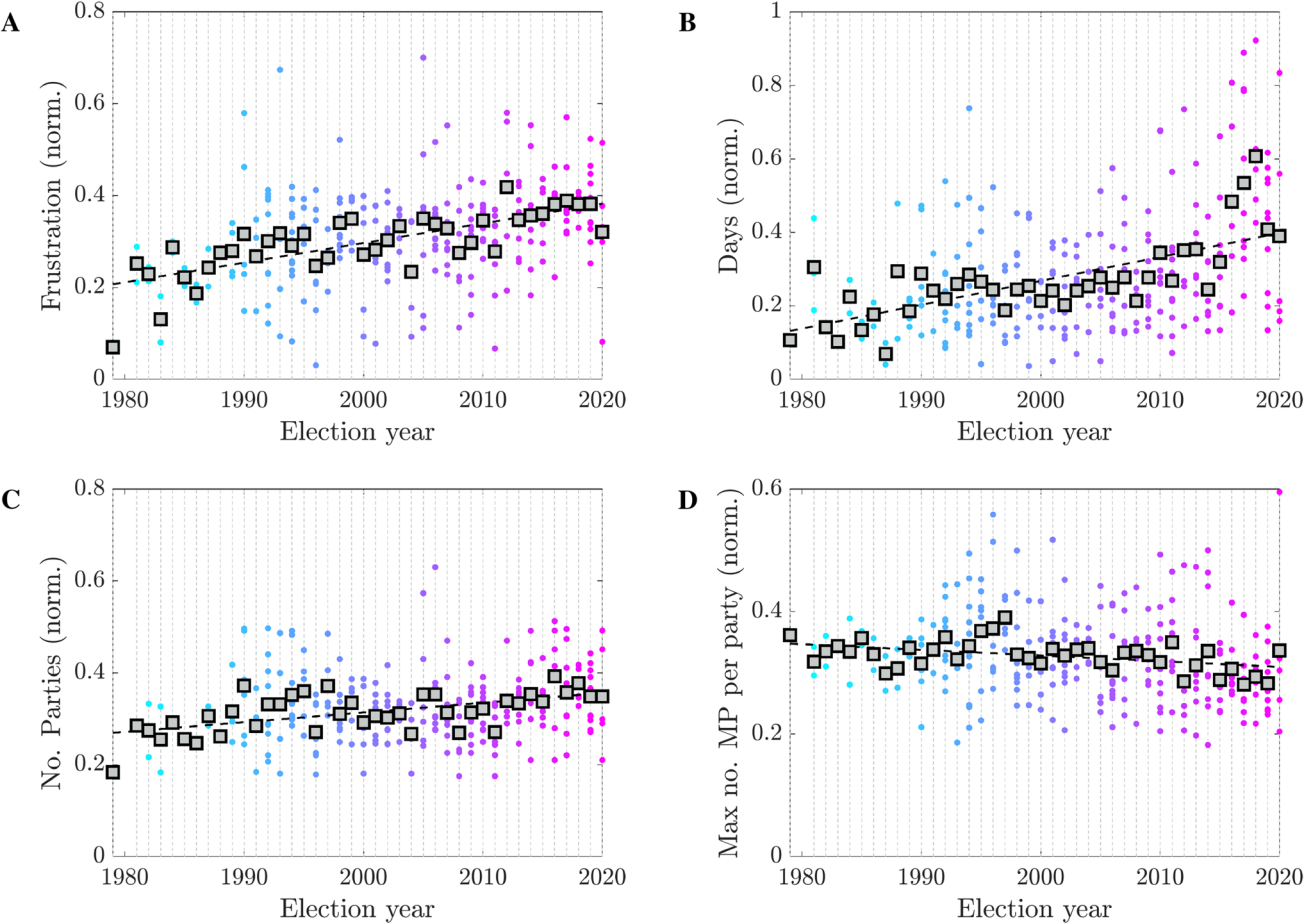
Yearly trends for the ensemble of 29 nations of Table 1 (normalized values). The gray boxes indicate the mean value for each year, the dashed black line the best linear fit for these mean values. (**A**) Frustration for scenario **I**; (**B**) duration of the government negotiation talks (days); (**C**) number of parties in parliament; (**D**) maximum number of MPs per party in parliament.

## Discussion

A cornerstone of parliamentary democracies is plurality of opinions, which often manifests itself as antagonism between parties of different factions. This is the *rationale* behind our use of the formalism of signed graphs for describing parliamentary networks and their collective decision processes. In particular, in the paper we investigate how our knowledge over signed parliamentary networks can be used in order to predict (1) the duration of the government negotiation talks and (2) the composition of the successful coalition cabinet formed after the elections. We show that the frustration of the parliamentary networks can be considered a good proxy for the complexity of the negotiations among the parties and, in particular, that it correlates well with the duration of the government negotiation talks. Moreover, we show that it can be used to obtain an estimate of the post-election government composition.

In the literature on political networks, signed networks have been used for instance to investigate coalition formation from policy beliefs^37–39^, to analyze the influence of political polarization on cabinet stability and duration^40^, or for inferring political polarization from voting records^41^. The approach we take in this paper is different as we use signed networks to investigate the government negotiation process, in particular the one that immediately follows a general election. It is known in the literature^4,6^ that the dynamics of such post-electoral government formation is somewhat simpler to analyze than that of inter-election governments. In fact, the antagonism among parties likely tends to peak during the pre-electoral period, hence it is natural that also the early phase of a legislation keeps reflecting the pattern of alliances and rivalries that characterized an electoral ballot. Furthermore, it is also plausible to assume that in early post-election decisions, MPs tend to follow thoroughly the party lines, and therefore that parties behave as homogeneous entities, as we are doing here. Modeling inter-election government processes may require to take into account the behavior of individual MPs and their history of votes, which requires to collect different datasets, difficult to obtain and even more difficult to analyze^41^.

If we zoom on the scatter plots of frustration vs negotiation days of the individual nations (see Fig. 3 for scenario **I**, Supplementary Fig. 5 and 6 for scenarios **II** and **III**), we see that in several cases what determines the high correlation is a small fraction of the data, corresponding to elections with a “hung parliament”. In our framework, rather than being considered spurious points, these data carry a strong informative value, as they correspond to parliamentary networks having a frustration higher than usual for that nation (in Fig. 3: a right outlier on the horizontal axis, according to a leverage statistic test). In the vast majority of cases, they happen to correspond to long cabinet negotiation times, i.e., to outliers from above also in the vertical axis (residual analysis test), see for example the plots for Germany, Ireland, Spain and Sweden in Fig. 3.

The Czech Republic seems to be the only exception to this rule, with a high value of negotiation days for the 2006 election corresponding to the least frustration. As a matter of fact, this point represents a special situation that does not violate the general rule proposed in the paper. In fact, the 2006 Czech election saw a parliament split into two exact halves, which also corresponds to our $S_{\text {best}}$ having 50% of $+1$ and 50% of $-1$. Since a tie is not a majority, its frustration may fail to capture the true complexity of the negotiation, if the two halves correspond also to opposite ideological factions, as in the Czech case. More precise comments on this example are reported in the “Supplementary Information”.

In the Czech Republic constitution, a cabinet is first sworn in and then confirmed by a confidence vote within 30 days. In two occasions (after the 2006 and 2017 elections) the candidate cabinets (Topolánek I and Babiš I) failed to obtain the confidence of the Parliament and new rounds of government negotiations started, after which the new candidate cabinets (Topolánek II and Babiš II) were sworn in. In our model, the 2006 and 2017 Czech elections give rise to two points, one corresponding to failure and the other to success, see Fig. 3 and Supplementary Fig. 7A.

The Czech unsuccessful attempts are among the few cases of “failure” in our datatset. In fact, in the 29 countries we analyzed, restricting to the post-election phase, only in a few cases unsuccessful (“official”) confidence votes have occurred and are therefore available for our data analysis (the countless failed attempts of formateurs normally stop without a formal vote, and are impossible to document systematically). Other failure points can be obtained if we consider legislatures that ended without a government being sworn in. We could find such data for Spain and Greece, see “Supplementary Information” and Supplementary Fig. 7A for more details. Figure 3 shows the correlation between frustration and duration of the government negotiation talks with and without these failure points. It can be seen that our predictions improve when also the latter are included.

Alongside the Czech case, in the “Supplementary Information” we analyze in detail several cases in which a hung parliament led to long negotiation times (Ireland in 2016/2020, Spain in 2015/2016/2019 and Germany in 2017). Here it is instructive to give some detail for the Swedish elections of 2018. In Sweden after the 2018 elections it took a record four months to form a government, and in fact the 2018 point has the highest frustration and is highly influential in the regression line. This example is also emblematic of the difficulties encountered when taking into account a priori information like pre-electoral coalitions or ideological classifications of parties (i.e., rile). The government formed after the 2018 Swedish elections, Social Democrats (S) and Green Party (MP) with support in the form of abstention from Centre Party (CP), Liberals (L) and Left Party (V), broke the pre-electoral coalition alliances (S+MP+V, Moderate Party+CP+Christian Democrats+L) hence in scenario **II** the 2018 election point is still influential, but leads to an overall negative correlation, see Supplementary Fig. 5. On the other hand, for certain countries adding coalition and ideological spectrum information increases significantly the correlation. For instance in Italy it increases from 0.06 to 0.45 (scenario **II**), which grows further to 0.95 in scenario **III**, see Supplementary Figs. 5 and 6.

Under the assumption of homogeneous party behavior, the adjacency matrices describing the parliamentary networks have blockwise identical entries, see “Supplementary Information” for the details. This means that the energy landscape determined by the functional in Eq. (1) with spin-like (i.e., $\pm 1$) states can be explored systematically. For scenario **I**, the values of energy corresponding to the various configurations are shown in Supplementary Fig. 3 for all countries and all elections. Since the weights in this scenario are restricted to $+1$ and $-1$, there are normally several degenerate ground states (leftmost bin in the histograms of Supplementary Fig. 3, see also Supplementary Fig. 8), as well as many other low energy states. Each state corresponds to a partition of the parliament into two aggregations of parties. The index $\eta _{\text {gov}}$ shown in Fig. 2C expresses how close the energy of the “true government” is to that of the ground state. As can be seen in Supplementary Fig. 3, in the vast majority of cases, it is very close to the ground state energy ($\eta _{\text {gov}}=0.95$ in scenario **I**). When the adjacency matrix is weighted (scenario **II** and **III**) degenerate states become less frequent. The value of $\eta _{\text {gov}}$ decreases, although it remains always around 0.8, sign that the “true government” is never “energetically implausible” for our selected weights (i.e., formed by implausible alliances of ideologically distant parties, unless this is strictly necessary for a particular parliament to achieve a majority).

*En passant* it is worth observing that our candidate government coalition $S_{\text {best}}$ subsumes also the notion of “minimal winning coalition” of^1^, i.e., in the ground state a government majority is reached but not unnecessarily exceeded. This is always true for scenario **I**, while in scenarios **II** and **III** the government coalition can be minimal or surplus, depending on the party coalitions.

The fractionalization index *F* (see “Methods” for a definition) is a widely used measure of concentration^11^. In our context, it is linked to the probability that two randomly chosen MPs belong to different parties. The equally popular effective number of parties $N_2$^10^ can be derived straightforwardly from it: $N_2 =1/(1-F)$. The “power 2” present in the formulas for *F* and $N_2$ (see “Methods”) has been interpreted for instance in terms of mean and variance of the distribution of seats per party^42^. By looking from a signed network perspective, for scenario **I**, we can give another interpretation, namely in terms of number of positive/negative edges associated to the parliamentary network. In fact, *F* is the fraction of negative edges in the signed graph, and for scenario **I** the frustration follows very closely this index, see Fig. 2D. The only cases in which it deviates slightly (e.g. Albania, North Macedonia, UK, see Supplementary Fig. 9) are associated to predicted minimum winning government coalitions $\mathcal {P}_{\text {best}}$ (corresponding to $S_{\text {best}}$) which consist of a number of seat in excess of the absolute minimum of 50% $+1$ of the seats. An exact formula expressing frustration in terms of fractionalization index and seat excess in a minimum winning coalition is given in the “Methods” (see also “Supplementary Information”). The consequence is twofold: first, even though frustration (a measure on signed graphs) is conceptually different from standard measures of parliamentary fragmentation, the fact that it nearly perfectly correlates with the fractionalization index *F* means that it is consistent with the existing literature. Second, as opposed to the classical fragmentation indexes, frustration also encodes information on the minimum winning coalition principle.

If in scenario **I** frustration is a close proxy for classical measures of parliamentary fragmentation, in scenarios **II** and **III** the two quantities $\zeta$ and *F* no longer coincide, as the signed adjacency matrix of a parliamentary network now has become weighted, which alters the value of $\zeta$, see Fig. 2D. In this respect, the method we are proposing is quite versatile, as it can easily incorporate both the basic fragmentation measure used in the literature as well as other factors, like ideological polarization and pre-existing party coalitions, as long as these factors can be expressed as sets of weights for the adjacency matrix of the signed parliamentary network.

In conjunction with the dynamical model shown in Fig. 1C, it is worth observing that crossing a bifurcation in our model (7) always leads to the onset of two equilibria with opposite signatures, say $x^*$ and $- x^*$. These two equilibria are aligned with the direction of the eigenvector of the least eigenvalue of the normalized Laplacian, and are related to success ($S_{\text {best}}$, which has more positive than negative entries) and failure ($- S_{\text {best}}$) of a confidence vote. In the vast majority of cases, only the first equilibrium is seen in our data, although some sporadic cases of failure to win parliamentary confidence (with a formal vote) are reported for some countries and are mentioned above. Our model (7) is symmetric and cannot account for this symmetry breaking effect. However, more sophistical models, such as the one considered in^28,29^, in which external influences and peer pressure lead to unfolding of the pitchfork bifurcation, can be used to capture this phenomenon. In particular, in the context of parliamentary systems, the pressure from the public opinion, from the media and from the parliament itself is to avoid a formal vote of confidence when the chances of passing it are null or even just slim, unless dictated by constitutional rules. How to modify the model (7) to account for these external factors will be the subject of future research.

In order to understand the confounding influence of unmodeled factors on our statistics, it is worth noticing that low values of $\rho _{\text {gov}}$ or $\eta _{\text {gov}}$ can often be observed in two special cases not explicitly included in our model: when a minority government is formed after the election (our government configuration $S_{\text {gov}}$ does not include parties that support the government in the parliament but have no assigned ministers) or, in bicameral systems, when a candidate cabinet needs to achieve a majority in both chambers of the parliament (only the lower chamber is analyzed in this study). Consider for example Italy: low values of $\eta _{\text {gov}}$ are obtained after the 1996, 2013 and 2018 elections, see scenario **II** and **III** in Fig. 5. The 1996 election sees the formation of a minority government, the Prodi I cabinet (a coalition of centre-left parties) which, while enjoying the confidence in the Senate of the Republic, did not manage to secure a majority in the Chamber of Deputies. This government had to be supported by the Communist Refoundation Party (PRC) and some other smaller parties in order to achieve majority in both chambers of the parliament. If we include these parties in the government configuration, now described by $S_{\text {gov}+\text {supp}}$ (see “Methods”), we can observe that the energy gap between the ground state and the energy functional $e(S_{\text {gov}+\text {supp}})$ decreases, see the 1996 election in Fig. 5. The opposite situation happened instead at the 2013 election where, while a clear majority was reached in the Chamber of Deputies by the centre-left alliance (that won 345 of the 630 seats), none of the party alliances obtained a majority in the Senate, whose seats were won mostly by three party blocs: the centre-left alliance, the centre-right alliance and the Five Star Movement (M5S), holding respectively 123, 117 and 54 of the 315 total seats. However, neither the centre-left nor the centre-right coalition wanted to form a government with the M5S party. After 62 days of government negotiations, the Letta cabinet, a grand coalition comprising parties from both the left and right side of the political spectrum, was sworn in. This configuration of parties was far from being a “minimum winning coalition” which, together with the fact that pre-electoral coalitions had to be broken to reach a cabinet coalition of ideologically distant parties, explains the low value of $\eta _{\text {gov}}$ we obtain for the 2013 election in all three scenarios. For example, in scenario **III** where the pre-electoral coalitions and the ideological differences between parties are taken into consideration, our ground state corresponds exactly to the centre-left coalition, which is energetically far from the Letta cabinet coalition, see Fig. 5 (2013 election). Moreover, both the 2013 and 2018 elections are examples on how the presence of the “big tent” M5S party (which does not have a clear classification on the left-right scale) further increases the complexity of the government negotiation process: in 2018 the Conte I cabinet was sworn in only after an agreement was reached between the far-right Lega and the M5S and, at 89 days, it is the longest government negotiation process since 1992 (where we start our analysis). The same conclusion applies if we consider the signed network corresponding to the Senate of the Republic. In all three scenarios the governing coalition (together with its support in the parliament) corresponds to the least energy achieving a majority in both upper and lower chamber, see Fig. 6 for scenario **III**, with the exception of the 1992, 2013 and 2018 elections.

There is a significant difference between scenarios **I** and **II** on the one hand, and scenario **III** on the other. In the first two scenarios, in fact, it is possible to claim that the correlation observed is actually a form of *causation*: the frustration of a network is computed solely on the basis of the data of the problem available *ex ante* (n. of parties and n. of MPs per party for scenario **I**; n. of parties and MPs plus coalition and rile for scenario **II**) without making use of any tuning parameter to fit the data. Hence a claim that the level of frustration is a “cause” (or one of the causes) behind the duration of the government negotiation phase, seems a reasonable one, given our interpretation of frustration as amount of antagonism present in a parliament. In scenario **III**, instead, the edge weights of the parliamentary network are tuned so as to maximize the correlation between frustration (a function of the edge weights) and duration of the negotiations, hence this scenario corresponds more to an *a posteriori* party classification based on a party behavior over the years. The rationale behind this approach is that a party “realpolitik” is a combination of factors such as those composing indexes like rile (ideological, social, economical, military, etc) but also of other variables like pragmatism, opportunity, necessity, national interest, etc, whose relative importance is difficult to assess and quantify. Notice that unlike in scenario **II** where new rile values are available at each new general election, in scenario **III** we keep the weights fixed throughout each party history, meaning that this method can have predictive power for future elections. To evaluate such predictive power, we can consider a “leave-the-last-one-out” validation, which is a particular case of the “leave-one-out” analysis we have performed (see Fig. 4 and “Methods”). When we consider the last (most recent) election as the validation set, we can observe that our model (scenario **III**) is able to predict the duration of the negotiations in most countries, see e.g., Denmark, Finland, Iceland, Italy, Slovakia, Spain, etc., in the regression plots of Supplementary Fig. 10. However, there are few cases in which our scenario **III** must necessarily fail, namely when a country experiences a hung parliament for the first time in the latest election, see Sweden or Germany in Supplementary Fig. 10. These failures are of course expected given the nature of the model and of the test (the validation set proposes a novel situation to the model). This confirms that high leverage and influential points are crucial to our approach.

As can be seen in Fig. 3, the scales for both frustration and government negotiation days vary widely between states. For the frustration this is due to factors such as number of MPs and parties, while for the negotiation days it is due to different national constitutional rules and traditions. Nevertheless, after a suitable normalization (see “Methods”), the data for the different nations can be assembled into pan-European time-series. As shown in Fig. 7, a few significant trends are clearly emerging: both frustration and government negotiation times have nearly doubled over the last 30 years. The increased frustration is due to an increase in parliamentary fragmentation (recall that in this scenario the frustration follows very closely the fractionalization index *F*), which is reflected by the increase in the number of parties represented in parliament, see Fig. 7C, and by parties of smaller size, see Fig. 7D. Likely factors behind these trends are the erosion of ideology-driven historical parties, the rise of populist and nationalistic parties and the appearance of post-ideological movements such as the “big tent” parties (e.g., M5S in Italy and United We Can in Spain). Overall, these factors add “disorder” to our parliamentary networks. In spite of the political sphere being commonly referred to as the quintessential “art of compromise”, this increased disorder puts a strain on the functioning of our political systems. By representing disorder in terms of graph frustration, our models provide a natural explanation and a quantitative assessment of some of the observable consequences of this phenomenon.

## Materials and methods

### Data description

A total of 29 European countries, whose system of government is a Parliamentary Republic or a Parliamentary Constitutional Monarchy, were considered in the analysis and are listed in Table 1. Countries adopting a Presidential (e.g., Belarus, Cyprus) or Semi-presidential (e.g., France, Georgia, Lithuania, Poland, Portugal, Romania, Russia, Ukraine) system were not considered. Countries that have switched from Semi-presidential to Parliamentary system in recent times (e.g., Armenia) were also disregarded. Countries with a short history of political elections (less than five) were excluded from the analysis (e.g., Montenegro, Kosovo). The Republic of Malta has not been considered given that there are only two parties competing in the elections, hence its parliamentary graph is always structurally balanced, with zero frustration (see below). When a bicameral system is adopted, we only consider data for the lower house.

We are interested only in general elections called after the dissolution of the parliament and only in the cabinet formation process immediately following the elections.

Data were collected from various sources such as the Manifesto Project Database^43^, the Parliaments and Governments Database^44^, the new Parline (IPU’s Open Data Platform)^45^, the Chapel Hill Expert Survey (CHES)^46,47^ and Wikipedia. To review the information other references were consulted, such as^48–52^ and^3^ (in particular the last reference provided additional information regarding the existence of pre-electoral coalitions, for a complete list of references see the “Supplementary Information”). The following data are considered: the election dates, the political parties winning seats at the elections and their position in the left–right political spectrum, the existing pre-electoral alliances, and the composition of the government formed after the elections and approved by the Parliament (confidence vote) in terms of swearing-in date, composition and status (minority or majority). Failures in forming a government are determined from unsuccessful confidence votes or from dissolutions of parliament without any government having been formed. All data were cross-checked on different datasets whenever possible.

As Table 1 shows, the number of elections considered for each country varies between 6 and 13, and we decided to start the analysis from election years around 1980 or later, given the difficulty to find information about the elections and/or parties involved before that year. However, some events considered to be particularly influential have moved the starting point for some countries, see Supplementary Table 1. Our data collection stops at Fall 2020. The data used in this study are available in the “Supplementary Data” files.

### Construction of parliamentary networks

A parliamentary network is an undirected, complete graph in which each MP is a node. MPs from the same party are linked through positive edges of weight equal to $+1$, while MPs from different parties are linked through signed edges that can be chosen according to the following scenarios (see Fig. 1A). **I**: unweighted all-against-all (all weights equal to $-1$), see Fig. 1B; **II**: weighted according to the ideological spectrum (computed according to the rile index^32,43^) and pre-electoral coalitions (MPs from parties involved in a coalition have positive edge weights), see Supplementary Fig. 1A (top); **III**: weighted according to both a semi-randomized (and optimized) left-right grid and pre-electoral coalitions, see Supplementary Fig. 1A (bottom). In this last case we considered 10,000 different sets of values for the party positions in the left–right political spectrum (which we kept fixed for all the elections) and computed the corresponding values of correlation between frustration and duration of government negotiation talks for each set. For each country the “optimal” choice of political positions corresponds to the one giving highest correlation. See “Supplementary Information” for the details.

We assume that parties are homogeneous entities: each MP relates (in terms of cooperation or antagonism) to other parties’ MPs according to his/her party line. The resulting signed adjacency matrices are consequently always block matrices, see Fig. 1B and “Supplementary Information” for more details.

### Measuring the frustration of signed parliamentary networks

The notion of structural balance^21,25^ captures the idea that it is possible to split a graph into two subgraphs such that all edges on each subgraph are positive, while all edges through the cut set that splits the graph are negative. In our parliamentary network it could represent a two-party parliament or, in a scenario including electoral coalitions, a parliament split into two coalitions. In general, the signed parliamentary networks we obtain (in all scenarios) are not structurally balanced. In this case, we use the notion of “frustration” to measure the distance of a signed network from a structurally balanced state. Let $\mathcal {L}$ be the normalized Laplacian corresponding to the signed graph, defined as $\mathcal {L}= I-(\text {diag}\{ |A |\mathbb {1}\})^{-1}A$, where *A* is the adjacency matrix of the network, $|\cdot |$ is the element-wise absolute value, $\mathbb {1}$ is the vector of 1s and $\text {diag}\{x\}$ is a diagonal matrix having the vector *x* on the diagonal. By associating a binary variable $s_i=\pm 1$ (spin up and down) to each party, one can define an energy-like quantity

1 $$\begin{aligned} e(S) = \frac{1}{2} \sum _{i,j\ne i} [\, |\mathcal {L} | + S\mathcal {L}S\,]_{ij}, \end{aligned}$$

where $S=\text {diag}\{S_1,\ldots ,S_{n_{\text {p}}}\}$ with $S_i = s_i I_{c_i}$ and $s_i=\pm 1$ ($i=1,\ldots ,{n_{\text {p}}}$), ${n_{\text {p}}}$ is the number of parties in the parliament and $c_i$, $i=1,\ldots ,{n_{\text {p}}}$, is the number of seats for the *i*-th party ($\sum _{i=1}^{{n_{\text {p}}}} c_i=n$ where *n* is the number of seats in parliament). Notice that $e(S) = e(-S)$. The frustration of the parliamentary network corresponds to the ground state energy, i.e., the minimal of this functional over all possible combinations of parties

2 $$\begin{aligned} \zeta = \min _{ \begin{array}{c} S=\text {diag}\{S_1,\ldots ,S_{{n_{\text {p}}}}\},\\ S_i = s_i I_{c_i},\,s_i=\pm 1 \end{array}} e(S). \end{aligned}$$

Denote $\pm S_{\text {best}}$ the diagonal matrices achieving the optimum in (2), with $S_{\text {best}}$ (“success”) having more $+1$ entries than $-S_{\text {best}}$ (“failure”).

### Frustration vs fractionalization index

The fractionalization index *F* is one of classical measures of parliament fragmentation^11^. It can be expressed in terms of the Laasko–Taagepera effective number of parties $N_2$ as $F = 1-\frac{1}{N_2}$, where $N_2$ is defined as

3 $$\begin{aligned} N_2 = \frac{1}{\sum _{i=1}^{{n_{\text {p}}}} \left( \frac{c_i}{n}\right) ^2 } = \frac{n^2}{\sum _{i=1}^{{n_{\text {p}}}} c_i^2}, \end{aligned}$$

with $c_i$ the size of the *i*th party, ${n_{\text {p}}}$ the total number of parties and *n* the total number of MPs^10^.

In our scenario **I** (all-against-all, no pre-electoral coalitions), the fractionalization index *F* corresponds to the fraction of negative entries in the adjacency matrix of the network. In fact, since all the party–party weights are equal to $-1$ and *A* is a $n\times n$ block matrix where each block has size $c_i \times c_j$ ($i,j=1,\ldots ,{n_{\text {p}}}$), it follows that the total number of negative elements in *A* is equal to $\sum _{i,j\ne i,=1}^{n_{\text {p}}}c_i c_j = n^2- \sum _{i=1}^{n_{\text {p}}}c_i^2 = n^2 \bigl (1- \frac{\sum _{i=1}^{n_{\text {p}}}c_i^2}{n^2}\bigr )= n^2 F$ and that the fraction of negative entries is equal to *F*.

As shown in Fig. 2D and Supplementary Fig. 9, the frustration of the unweighted signed networks of scenario **I** has a very high correlation (0.99 in average) with *F*. To explain this correlation, we can observe that the frustration of a signed unweighted parliamentary network corresponds (up to a multiplicative constant) to a difference of three terms, the fractionalization index *F*, a constant term and a third term interpretable as the “distance” of the minimum winning coalition in correspondence of $S_{\text {best}}$ from the 50% of the seats:

4 $$\begin{aligned} \zeta = \frac{n^2}{n-1} \cdot \left( F -\frac{1}{2} +\underbrace{\frac{1}{2}\left( \frac{E_{\text {best}}}{n/2}\right) ^2}_{\text {``distance'' of }\mathcal {P}_{\text {best}}\text { from }50\%} \right) , \end{aligned}$$

where $\mathcal {P}_{\text {best}}$ describes the majority coalition in correspondence of $S_{\text {best}}$ (see also next section “Minimum energy government coalition”) and $E_{\text {best}}\in [0,\frac{n}{2}]$ is the number of seats in excess of $\mathcal {P}_{\text {best}}$ with respect to 50% of the total number of seats: $\sum _{i:p_i\in \mathcal {P}_{\text {best}}} c_i = \frac{n}{2}+ E_{\text {best}}$, where $p_i$ represents the *i*th party (see also Supplementary Sect. S2.4 for more details). This “distance” attains its minimum value when $S_{\text {best}}$ corresponds to 50% $+1$ of the seats in a parliament, and grows when the seat excess corresponding to $S_{\text {best}}$ grows.

### Minimum energy government coalition

For each country and parliamentary election we compute the energy (1) for all $2^{n_{\text {p}}}$ (${n_{\text {p}}}$ is the number of parties in the parliament) possible party configurations *S* (see Supplementary Fig. 3 for the energy landscape in scenario **I**) and the frustration (2) of the signed parliamentary network as the minimum of such energies. Our predicted government coalition, denoted $\mathcal {P}_{\text {best}}$, is given by the corresponding group of parties achieving a majority in $S_{\text {best}}$: $\sum _{i:\,p_i\in \mathcal {P}_{\text {best}}} c_i >\frac{n}{2}$ where $c_i$ is the number of seats gained by the party $p_i$ and *n* is the total number of MPs in parliament. Similarly, let $S_{\text {gov}}$ be the party coalition that effectively obtained a confidence vote for that election and $\mathcal {P}_\text {gov}$ the list of corresponding parties, $S_{\text {gov}}= \text {diag}\{s_1 I_{c_1},\ldots , s_{n_{\text {p}}}I_{c_{n_{\text {p}}}}\}$ with $s_i=+1$ if $p_i \in \mathcal {P}_\text {gov}$ or $s_i = -1$ otherwise. Denoting the cardinality of a set as $\text {card}\left( \cdot \right)$, the fraction of government coalition captured by the ground state party coalition $\mathcal {P}_{\text {best}}$ can be defined as

5 $$\begin{aligned} \rho _{\text {gov}}= \frac{\text {card}\left( \mathcal {P}_{\text {best}}\,\cap \, \mathcal {P}_{\text {gov}}\right) }{\text {card}\left( \mathcal {P}_{\text {gov}}\right) }, \end{aligned}$$

see Fig. 2B (and Supplementary Fig. 2C, for scenario **I** only). The closer the value of $\rho _{\text {gov}}$ is to 1, the better our estimate represents the actual cabinet composition. In scenario **I** the ground state may be “degenerate”, i.e., multiple signature matrices $S_{\text {best}}$ could give the same value of frustration, and/or the corresponding party group $\mathcal {P}_{\text {best}}$ may hold exactly half of the seats in the parliament ($\sum _{i:\,p_i\in \mathcal {P}_{\text {best}}} c_i =\frac{n}{2}$). In the first case, we consider the matrix $S_{\text {best}}$ giving maximum value of $\rho _{\text {gov}}$. In the second, we take as $S_{\text {best}}$ the matrix that yields the least value in (2) while satisfying the condition $\sum _{i:\,p_i\in \mathcal {P}_{\text {best}}} c_i >\frac{n}{2}$ (see “Supplementary Information” for the details). The case in which $S_{\text {best}}$ is unique and its $\mathcal {P}_{\text {best}}$ corresponds to exactly $\frac{n}{2}$ is also degenerate as the ground state has no majority.

It is also possible to compare the frustration $\zeta$ with the actual government “energy”:

6 $$\begin{aligned} \eta _{\text {gov}}= 1 - \frac{e(S_{\text {gov}})-\zeta }{\max _S e(S)-\zeta }. \end{aligned}$$

$\eta _{\text {gov}}$ expresses how close the energy of the actual government is compared to the frustration, i.e., the theoretical minimum of such quantity over all possible party combinations, see Fig. 2C (and Supplementary Fig. 2D, for scenario **I** only).

When a minority government $\mathcal {P}_\text {gov}$ needs additional support to win a confidence vote, we denote $\mathcal {P}_\text {supp}$ the set of parties which support the cabinet in the parliament without being explicitly part of it and $S_{\text {gov}+\text {supp}}= \text {diag}\{s_1 I_{c_1},\ldots , s_{n_{\text {p}}}I_{c_{n_{\text {p}}}}\}$ the corresponding party configuration, with $s_i=+1$ if $p_i \in \mathcal {P}_\text {gov}\cup \mathcal {P}_\text {supp}$ or $s_i = -1$ otherwise. The intuition is that $e(S_{\text {gov}+\text {supp}})$, i.e., the value of energy functional corresponding to the (majority) coalition supporting the government in the parliament (computed according to (1)), should be energetically closer to our ground state than $e(S_{\text {gov}})$, i.e., the energy functional corresponding to the (minority) government. See for example the 1996 election in Italy in Fig. 5. This analysis is carried out only for Italy, however we expect similar results for the other countries where minority governments are common, such as Denmark, Norway or Sweden (see Supplementary Fig. 7B).

### Dynamical model of government formation

We can describe the government formation process by the nonlinear interconnected model of collective decision-making introduced in^29–31^,

7 $$\begin{aligned} \dot{x} = -\Delta x +\pi A\psi (x),\quad x\in \mathbb {R}^n. \end{aligned}$$

In (7), *n* is the number of elected MPs, the vector $$x=\begin{bmatrix} x_1&\cdots&x_n\end{bmatrix}^T$$ represents their opinions, *A* is the adjacency matrix of the signed parliamentary network, $\Delta =\text {diag}\{\delta _1,\ldots ,\delta _n\}$ is a diagonal matrix such that $\delta _i=\sum _{j\ne i} |a_{ij} |$ for all *i*, $$\psi (x)=\begin{bmatrix} \psi _1(x_1)&\cdots&\psi _n(x_n)\end{bmatrix}^T$$ is a vector of sigmoidal and saturated nonlinearities (expressing how the MPs transmit their opinion to the other MPs in the network) and $\pi$ is a positive scalar parameter acting as bifurcation parameter. More precisely, the bifurcation occurs at

8 $$\begin{aligned} \pi _1=\frac{1}{1-\lambda _1(\mathcal {L})}, \end{aligned}$$

see Fig. 1C, where $\lambda _1(\mathcal {L})$ is the smallest eigenvalue of the normalized signed Laplacian $\mathcal {L}$ associated to (7), see “Supplementary Information” for the details.

### Rationale behind the correlation between frustration and duration of the government negotiation phase

If we denote $\tau$ the duration of the government negotiation phase, from election day to the day the government is sworn in (and, for the cases of failure, to the day in which the formateur looses a confidence vote or to the day in which the parliament is dissolved without a government having been formed), then $\tau$ and the frustration $\zeta$ are related by a form of direct proportionality (here indicated by the symbol “$\sim$”): $\tau \sim \zeta$. This can be explained in terms of the following chain of relationships linking $\tau$ to $\zeta$ via the bifurcation parameter $\pi _1$ and the algebraic conflict $\lambda _1(\mathcal {L})$:

$$\begin{aligned} \tau \sim \pi _1 , \qquad \pi _1 = \frac{1}{1-\lambda _1 (\mathcal {L})}, \qquad \lambda _1 (\mathcal {L}) \sim \zeta \end{aligned}$$

While the second on these relationships is analytical, the first and third are only numerical. The correlation plots of Fig. 3 (Supplementary Figs. 5 and 6 for scenario **II** and **III**, respectively) are an expression of this $\tau \sim \zeta$ relationship.

### Leave-one-out analysis for scenario **III**

In scenario **III**, differently from **I** and **II**, the political positions of the parties are fixed in time and are chosen to maximize the Pearson correlation between frustration of the parliamentary networks and duration of the government negotiations. To validate this method we have performed a leave-one-out analysis, whose idea is to use only $N-1$ elections (where *N* is the number of elections for each country) to determine the political positions of each party and then check how well the model “fits” the excluded election, by comparing the Pearson correlation coefficients (frustration of the parliamentary networks v.s. duration of the government negotiations) obtained with and without the data point corresponding to this election.

The procedure is described in detail as follows. For each country, the data on the parliamentary elections are divided into two sets: a validation set, made of a single election, and a training set, made of the remaining ($N-1$) elections. The training set is used to find the “optimal” choice of political positions by maximizing the correlation frustration v.s. duration of the government negotiation talks. 1000 sets of values for the left-right political positions of the parties (see Eq. (S2) in the “Supplementary Information”) are randomly selected on the preassigned left-right grid, and for each choice of political positions the Pearson correlation (frustration v.s. days) is calculated: the “optimal” choice for the weights corresponds to the one giving the best correlation index (among the 1000). This choice for the weights is used to build the parliamentary network corresponding to the excluded election (the validation set), whose frustration is then computed. Finally, the correlation (frustration v.s. days), when all the *N* elections are considered, is calculated. This process is repeated *N* times, each time by selecting a different election as validation set, obtaining (for each country) $N\times 1000$ correlation indexes calculated using the training sets and *N* “optimal” choices for the weights and corresponding values of correlation.

The values of correlation (frustration v.s. days) obtained when optimizing for $N-1$ elections (i.e., leave-one-out analysis) are then compared with those obtained when optimizing for *N* elections (i.e., scenario **III**). Figure 4 illustrates these results. A particular case of this analysis is the “leave-the-last-one-out” validation, where the validation set consists of the last election. Supplementary Fig. 10 shows the regression plots between duration of government negotiations and frustration where the weights of the parliamentary networks are chosen so as to maximize the correlation (days v.s. frustration) in the first $N-1$ elections.

### Yearly trends

To obtain the yearly trends shown in Fig. 7 we compute and plot, for each country, the normalized values $\frac{x}{ \Vert x \Vert _2}$ over the election years, where *x* is the *N*-dimensional vector of interest (frustration, government negotiation days, number of parties in the parliament and maximum number of MPs per party), *N* is the number of elections, and $\Vert \cdot \Vert _2$ the Euclidean norm.

## Supplementary information

Supplementary Information 1.

Supplementary Information 2.

Supplementary Information 3.

Supplementary Information 4.

## Data Availability

All results are reported in the main text and “Supplementary Information”. The data used in this study are available in the “Supplementary Data” files.
