# Supplementary material for: A signed network perspective on the government formation process in parliamentary democracies
Source: Sci Rep. 2021 Mar 4;11:5134. doi: 10.1038/s41598-021-84147-3 (PMC7933210; doi:10.1038/s41598-021-84147-3)
Supplement: Supplementary file 1 — Supplementary Information 1. [file 41598_2021_84147_MOESM1_ESM.pdf]

Supplementary Data (legend) for

A signed network perspective on the government formation process in parliamentary democracies

Angela Fontan and Claudio Altafini*

*Division of Automatic Control, Department of Electrical Engineering, Linköping University
SE-58183 Linköping, Sweden, E-mail: {angela.fontan, claudio.altafini}@liu.se

Variable	Meaning
Country	Name of the country.
DateElection	Date (day, month, year) of general election.
DateFailure	Government negotiations failure date (day, month, year), determined for the Czech Republic as the date the cabinet (which later failed to pass the investiture vote) was formed, for Greece as the date the caretaker cabinet was sworn in, and for Spain as the date corresponding to two months after the first vote for investiture.
DateGovernment	Sworn in date (day, month, year) of the government formed after the election.
Parties	Abbreviations of parties winning seats at the election.
Seats	Number of seats won by each party at the election.
Government	Binary variable indicating if a party belongs to the government formed after the election (0 = no, 1 = yes).
PoliticalPosition	(scenarios **II** and **III** only) Political position of a party in the left-right spectrum.<br>**II**: the political position is given by the rile value, and it belongs to the interval [-0.5,0.5]. See Supplementary Information for more details.<br>**III**: the political position is classified as one of the following: far-left, left to far-left, left, centre-left to left, centre-left, centre to centre-left, centre, centre to centre-right, centre-right, centre-right to right, right, right to far-right, far-right, big tent.
PEC_i	For each pre-electoral coalition i, the binary variable PEC_i indicates if a party participates to the pre-electoral coalition (0 = no, 1 = yes).

Table 1: Legend of Supplementary Data 1, showing the data used in this study. Each worksheet in the .xlsx file corresponds to a scenario, **I**, **II**, **III**.

Variable	Meaning
Country	Name of the country.
DateElection	Date (day, month, year) of general election.
days_failure	Government negotiation days (including the elections corresponding to failure of the government negotiations).
frustration_failure	Frustration of the signed parliamentary network for each country and election, i.e., ζ (including the elections corresponding to failure of the government negotiations).
correlation_failure	Pearson's correlation index between the government negotiation days and the frustration (one value for each country) (including the elections corresponding to failure of the government negotiations).
days	Government negotiation days.
frustration	Frustration of the signed parliamentary network for each country and election, i.e., ζ.
correlation	Pearson's correlation index between the government negotiation days and the frustration (one value for each country).
rhoGov	Index ρ_{gov} representing the overlap between the party coalition that succeeded in forming a government and the majoritarian group of parties in the ground state.
etaGov	Index η_{gov} representing how close "energetically" our guess (i.e., $e(S_{\text{best}})$) is to the true government energy $e(S_{\text{gov}})$.
rhoGov_Mean	Mean value of the variable rhoGov (one value for each country).
etaGov_Mean	Mean value of the variable etaGov (one value for each country).

Table 2: Legend of Supplementary Data 2, showing the results obtained in this study. Each worksheet in the .xlsx file corresponds to a scenario, **I**, **II**, **III**.
