# Supplementary material for: A signed network perspective on the government formation process in parliamentary democracies
Source: Sci Rep. 2021 Mar 4;11:5134. doi: 10.1038/s41598-021-84147-3 (PMC7933210; doi:10.1038/s41598-021-84147-3)
Supplement: Supplementary file 2 — Supplementary Information 2. [file 41598_2021_84147_MOESM2_ESM.pdf]

Supplementary Information for
A signed network perspective on the government formation process in parliamentary democracies

Angela Fontan and Claudio Altafini[*]

[*]Division of Automatic Control, Department of Electrical Engineering, Linköping University
SE-58183 Linköping, Sweden, E-mail: {angela.fontan, claudio.altafini}@liu.se

S1 Methods

Symbol	Meaning
N	number of elections for each country.
n	number of elected parliamentary members for each election.
$\mathcal{G}$	signed network.
$\mathcal{G}_{\text{country, date}}$	parliamentary network for a country and election year "date".
$A = [a_{ij}]$	$n \times n$ adjacency matrix of $\mathcal{G}_{\text{country, date}}$.
n_{p}	number of elected political parties for each election.
p_i	i-th parliamentary political party, $i = 1, \ldots, n_{\text{p}}$.
c_i	number of seats gained by the i-th party p_i, $i = 1, \ldots, n_{\text{p}}$; it holds that $\sum_{i=1}^{n_{\text{p}}} c_i = n$.
q_{p_i}	"political position" of the i-th party p_i, $i = 1, \ldots, n_{\text{p}}$, in a left-right axis.
w_{ij}	weight (= amount of trust/distrust) between two parties p_i, p_j, respectively, $i, j = 1, \ldots, n_{\text{p}}$.
$W = [w_{ij}]$	$n_{\text{p}} \times n_{\text{p}}$ matrix of weights.
$\mathbb{1}_{c_i}$	$c_i \times 1$ vector of ones.
I_{c_i}	$c_i \times c_i$ identity matrix.
$E_{c_i c_j}$	$E_{c_i c_j} = \mathbb{1}_{c_i} \mathbb{1}_{c_j}^T$, $c_i \times c_j$ matrix of ones; notation simplified to E_{c_i} if $j = i$.
ϵ	frustration index.
ζ	party-wise frustration index.
S	signed diagonal matrix of ± 1.
$e(S)$	energy of the configuration state S.
$\mathcal{L}$	normalized signed Laplacian.
$\lvert\mathcal{L}\rvert$	element-wise absolute value of the normalized signed Laplacian $\mathcal{L}$.
$\lvert H\rvert$	element-wise absolute value of the normalized interaction matrix H.
π	bifurcation parameter.

S1.1 Parliamentary network construction

For each country and parliamentary election listed in Supplementary Table 1.we consider an undirected graph $\mathcal{G}_{\text{country, date}} = (\mathcal{V}, \mathcal{E}, A)$, where $\mathcal{V}$ (card $(\mathcal{V}) = n$) is the vertex set, $\mathcal{E}$ is the edge set, and $A = [a_{ij}] \in \mathbb{R}^{n \times n}$ is the adjacency matrix, with a_{ij} representing the weight of the edge $(j, i) \in \mathcal{E}$. Each of the n vertices

in $\mathcal{V}$ represents an elected Member of the Parliament (MP), and each edge in $\mathcal{E}$ the relationship between two MPs, which can be cooperative or antagonistic. A positive edge $a_{ij} > 0$ means cooperation between the i-th and j-th MPs, a negative edge $a_{ij} < 0$ antagonism. All MPs of a party are always assigned the same weights, i.e., we treat a party as a homogeneous cluster of nodes.

As shown in Fig. 1B in the main text, the network $\mathcal{G}_{\text{country, date}} = (\mathcal{V}, \mathcal{E}, A)$ is therefore composed of n_{p} clusters, represented by the political parties, where n_{p} is the total number of parties which gained seats in the parliament after the election. This implies that the adjacency matrix A, as well as all the other matrices of interest, can be seen as a $n_{\text{p}} \times n_{\text{p}}$ block matrix:

$$A = \begin{bmatrix} A_{11} & \dots & A_{1n_{\text{p}}} \\ & \ddots & \\ A_{n_{\text{p}}1} & \dots & A_{n_{\text{p}}n_{\text{p}}} \end{bmatrix}, \qquad \text{with} \quad A_{ij} = \begin{cases} (E_{c_i} - I_{c_i})\, w_{ii}, & j = i \\ E_{c_i c_j}\, w_{ij}, & j \neq i, \end{cases} \tag{S1}$$

where c_i is the number of seats gained by the i-th party, $E_{c_i c_j} = \mathbb{1}_{c_i} \mathbb{1}_{c_j}^T$ (simplified to E_{c_i} if $j = i$) is the matrix of all 1s ($\mathbb{1}_{c_i}$ is the vector of size c_i having all elements equal to 1), and $W = [w_{ij}] \in \mathbb{R}^{n_{\text{p}} \times n_{\text{p}}}$ is the matrix of party-party weights. Its signed entries w_{ij} describe the interaction between MPs of the party p_i and party p_j, in terms of political affinity.

In order to choose the matrix W, we consider different party grouping criteria and different weight assignment methods. The party grouping criteria are:

1. **All-against-all**. All parties compete against all parties:

$$w_{ij} \begin{cases} = 1 & \text{if } i = j \text{ } (p_i \text{ and } p_j \text{ are the same party; hereafter: } p_i = p_j) \\ < 0 & \text{if } p_i \text{ and } p_j \text{ are different parties.} \end{cases}$$

   Electoral coalitions are not taken into account in this case. Germany is an exception to this rule, in that the Christian Democratic Union of Germany (CDU) and Christian Social Union in Bavaria (CSU) are always considered as a single party.

2. **Pre-electoral coalitions**. Parties in pre-electoral coalitions are cooperating:

$$w_{ij} \begin{cases} = 1 & \text{if } p_i = p_j \\ > 0 & \text{if parties } p_i \text{ and } p_j \text{ belong to the same coalition} \\ < 0 & \text{if parties } p_i \text{ and } p_j \text{ do not belong to the same coalition.} \end{cases}$$

   Pre-electoral coalitions (explicit or implicit) are obtained from references and datasets such as (*1*–*6*), WIKIPEDIA and the new Parline (*7*).

   In the case of countries where double rounds of elections are common (for example in Hungary until 2010), we consider also the electoral coalitions made before the second round (so the ones made before the first round plus the ones made between the first and second round).

We always assume that $w_{ii} = 1$ and that $w_{ij} = w_{ji}$, i.e., that W (and hence A) is symmetric. In turn, this means that $\mathcal{G}_{\text{country, date}}$ is undirected.

As for the weights themselves, we consider both the cases of unweighted (but signed) W and weighted (and signed) W. In the **unweighted case**, $w_{ij} \in \{-1, +1\}$, with $+1$ only on the diagonal (all-against-all case). The resulting graph is complete, undirected and signed.

In the **weighted case**, the general philosophy is that off-diagonal weights between parties which are not in pre-electoral coalition should be negative, small (in absolute value) for ideologically close parties

and approaching -1 for ideologically antipodal parties. Instead, off-diagonal weights between parties in pre-electoral coalition should be positive and close to 1 for ideologically close parties. In order to define the matrix of weights $W = [w_{ij}]$, the first step consists in assigning to each political party a position in the left-right ideological spectrum. For this, we follow two different criteria, as shown in Supplementary Fig. 1A: (1) use data from the Manifesto Project Database, in particular the so-called *rile* index (*8*); and (2) place the parties on a predetermined left-right grid and assign to them specific positions randomly. It is convenient to start the description by the second approach.

2. **Predetermined left-right grid with randomly assigned positions.** For each country and each political election, we consider each political party gaining seats in the Parliament and classify its political position as one of the following: Far-Left (FL), Left to Far-Left (LFL) , Left (L), Centre-Left to Left (CLL), Centre-Left (CL), Centre to Centre-Left (CCL), Centre (C), Centre to Centre-Right (CCR), Centre-Right (CR), Centre-Right to Right (CRR), Right (R), Right to Far-Right (RFR), Far-Right (FR), Big Tent (BT). Each of these labels X (except BT) occupies an ordered position q_{X} in the interval $[q_{\mathrm{FL}}, q_{\mathrm{FR}}] = [-0.5, 0.5]$, i.e., we have the following grid of ordered coarse-grained political positions between FL and FR:

$$\left[\; q_{\mathrm{FL}} \quad q_{\mathrm{LFL}} \quad q_{\mathrm{L}} \quad q_{\mathrm{CLL}} \quad q_{\mathrm{CL}} \quad q_{\mathrm{CCL}} \quad q_{\mathrm{C}} \quad q_{\mathrm{CCR}} \quad q_{\mathrm{CR}} \quad q_{\mathrm{CRR}} \quad q_{\mathrm{R}} \quad q_{\mathrm{RFR}} \quad q_{\mathrm{FR}} \;\right], \tag{S2}$$

see also Supplementary Fig. 1B.

We assume that the left-right scale is symmetric around the central position $q_{\mathrm{C}} = 0$. The specific values of q_{CCR}, q_{CR}, q_{CRR}, q_{R}, q_{RFR} are chosen randomly, sorting 5 values drawn from a uniform probability distribution in the interval $[0,\ 0.5]$. The values of q_{LFL}, q_{L}, q_{CLL}, q_{CL} and q_{CCL} are then obtained by symmetry, see Supplementary Fig. 1B. Big tent parties do not fit into such a left-right grid, because they tend to attract voters from the entire ideological spectrum. Examples of big tent parties are the Italian party *Movimento 5 Stelle* (5 Star Movement), in the 2018 elections. Consequently, the distance d_{BT} between a big tent party and any other party must not be "too small" nor "too big" compared with all the possible differences $|q_{p_i} - q_{p_j}|$ for all non big tent parties p_i and p_j: d_{BT} is then chosen as the median of the differences, taken in absolute value, between all possible combinations of the pairwise distances of the positions $q_{\mathrm{FL}}, \ldots, q_{\mathrm{FR}}$,

$$d_{\mathrm{BT}} = \text{median}\{|q_{\mathrm{FL}} - q_{\mathrm{LFL}}|, |q_{\mathrm{FL}} - q_{\mathrm{L}}|, \ldots, |q_{\mathrm{FL}} - q_{\mathrm{FR}}|, \ldots, |q_{\mathrm{RFR}} - q_{\mathrm{FR}}|\}. \tag{S3}$$

With these conventions we can proceed to assigning numerical values to the weights.

Since we take into account also pre-electoral coalitions, we need to assign weights that are positive to parties in the same coalition. The weights w_{ij}, $i, j = 1, \ldots, n_{\mathrm{p}}$, are then chosen with the following rule:

$$w_{ij} = \begin{cases} 1, & p_i = p_j \\ & \text{(same party)} \\ \text{coal}(p_i, p_j) - \dfrac{1}{2} \cdot \min\limits_{\substack{\mathrm{X,Y} \in \{\mathrm{C,CCR,\ldots,FR}\} \\ \text{s.t. } q_{\mathrm{X}} \neq q_{\mathrm{Y}}}} |q_{\mathrm{X}} - q_{\mathrm{Y}}| & p_i \neq p_j,\ q_{p_i} = q_{p_j} \\ & \text{(different parties, but placed on the same position)} \\ \text{coal}(p_i, p_j) - |q_{p_i} - q_{p_j}|, & p_i \neq p_j,\ q_{p_i} \neq q_{p_j},\ p_i \text{ and } p_j \text{ not big tent} \\ & \text{(different parties on different positions,} \\ & \quad \text{none is big tent)} \\ \text{coal}(p_i, p_j) - d_{\mathrm{BT}}, & p_i \neq p_j,\ q_{p_i} \neq q_{p_j},\ p_i \text{ or } p_j \text{ big tent} \\ & \text{(different parties on different positions,} \\ & \quad \text{one of them is big tent)} \end{cases} \tag{S4}$$

The function coal(p_i, p_j) is equal to 1 if p_i and p_j are in electoral coalition, 0 otherwise. Notice that when two parties p_i and p_j are not in electoral coalition but are located in the same position (i.e., $q_{p_i} = q_{p_j}$), then we still assume that the w_{ij} weight is negative, but small (equal to half the least nonzero difference between any two parties). The weights are kept constant throughout the party history.

To study how different choices of the values q_X can influence our analysis, we decided to consider 10000 different vectors of random values for each country, retaining the best value (i.e., the value that leads to the highest correlation between frustration and negotiation days, see below for more details).

1. **Rile index from Manifesto Project Database.** For each country and parliamentary election, the Manifesto Project Database (*9*) collects information on the electoral manifestos of the parties. The index denoted *rile* (*8*) summarizes their ideology according to various criteria (e.g., its position on economy, military, international relations, education, welfare, etc.). The political position of party p_i is given by the rile value of the party, properly rescaled to fit our $[-0.5, 0.5]$ normalization: $q_{p_i} = \text{rile}(p_i)$. In case of missing rile for a party p_i, its position q_{p_i} is determined using the method discussed above, only considering a vector (S2) which is equispaced in $[-0.5, 0.5]$ (i..e, the random assignment to the q_X value is skipped). The weight matrix W is then given by the formula (S4).

In the paper we consider three different combinations of edge weight assignments and party grouping methods listed in Fig. 1A in the main text.

S1.2 Structural balance and frustration for signed networks

Consider a signed, undirected, simple and connected network $\mathcal{G} = (\mathcal{V}, \mathcal{E}, A)$. The *normalized signed Laplacian* of $\mathcal{G}$ is defined as $\mathcal{L} = I - \Delta^{-1}A$ where $\Delta = \text{diag}\{\delta_1, \ldots, \delta_n\}$ has elements $\delta_i = \sum_{j=1}^{n} |a_{ij}| > 0$, $i = 1, \ldots, n$.

A signed network is *structurally balanced* if all its cycles are positive, i.e., if each cycle contains an even number of negative edges: in a social network context, every length-3 signed cycle of a structurally balanced network describes one of the following concepts: "the friend of my friend is my friend", "the enemy of my friend is my enemy", "the friend of my enemy is my enemy", "the enemy of my enemy is my friend", see (*10*) for more details. The notion of structural balance captures the idea that it is possible to split a graph into two subgraphs such that all edges on each subgraph are positive, while all edges through the cut set that splits the graph are negative. In our parliamentary network it could represent a two-party parliament or, in the electoral coalition scenario, a parliament split into two coalitions. Equivalent conditions to structural balance are (i) $\lambda_1(\mathcal{L}) = 0$, and (ii) there exists a signature matrix $S = \text{diag}\{s_1, \ldots, s_n\}$, with $s_i = \pm 1$, such that $S\mathcal{L}S$ has all nonpositive off-diagonal entries (*11*). It follows that a network $\mathcal{G}$ is structurally unbalanced if and only if $\lambda_1(\mathcal{L}) > 0$.

When a network is not structurally balanced, it is of interest to understand how "far" it is from a structurally balanced state. One idea is to use $\lambda_1(\mathcal{L})$ (the so-called "algebraic conflict" (*12, 13*)), which is strictly positive for structurally unbalanced networks, to measure such a distance; however, in the literature another standard measure, called *frustration index*, is more frequently adopted (*10, 14, 15*). It is defined as the minimum (weighted, if $\mathcal{G}$ is a weighted graph) sum of the positive edges over all signature similarity transformations of $\mathcal{L}$, $S\mathcal{L}S$, with S signature matrix (*16*):

$$\epsilon = \min_{\substack{S=\text{diag}\{s_1,\ldots,s_n\} \\ s_i=\pm 1}} \frac{\sum_{i,j\neq i}[|\mathcal{L}| + S\mathcal{L}S]_{ij}}{2}. \tag{S5}$$

The computation of ϵ constitutes a NP-hard problem: however, the intuition is that $\lambda_1(\mathcal{L})$ approximates well (up to a scaling factor) the value of the frustration index, and in particular that $\lambda_1(\mathcal{L})$ grows linearly with ϵ (*16*). Hence, they can both be used to measure the structural imbalance of a signed network.

The frustration index, as defined in (S5), is also the minimum of a weighted energy functional over all possible signature matrices S. This terminology is inherited from Statistical Physics, where a (unweighted) signed graph is interpretable as an Ising spin glass, and the various spin configurations ("spin up" and "spin down" at the nodes) determine the energy of the spin glass. The least energy that can be achieved by any configuration (called the ground state) corresponds to the frustration index (S5). The definition of energy functional introduced in (*10*) for this purpose can be adapted to networks that are weighted. The *weighted* energy functional can be defined as follows,

$$e(S) = \frac{\sum_{i,j}(|h_{ij}| - h_{ij}s_i s_j)}{2}, \tag{S6}$$

where S is a signature matrix, $S = \text{diag}\{s_1, \ldots, s_n\}$ with $s_i = \pm 1 \ \forall \ i$, and $H = [h_{ij}] = \Delta^{-1}A$ is the normalized adjacency matrix. Notice that $e(S) = e(-S)$. Notice further that by definition of normalized signed Laplacian, it follows that

$$e(S) = \frac{\sum_{i,j}[|H| - SHS]_{ij}}{2} = \frac{\sum_{i,j\neq i}[|\mathcal{L}| + S\mathcal{L}S]_{ij}}{2}.$$

Hence, the weighted frustration index is the minimum of the weighted energy functional over all possible signature matrices S (i.e., again, the ground state):

$$\epsilon = \min_{\substack{S=\text{diag}\{s_1,\ldots,s_n\} \\ s_i=\pm 1}} e(S).$$

As described above, each network $\mathcal{G}_{\text{country, date}}$ can be partitioned into n_p clusters (the political parties), with n_p being the number of parties involved and gaining seats in the elections, making the adjacency matrix A a $n_\text{p} \times n_\text{p}$ blocks matrix. Under the assumption that all MPs of a party follow loyally and unanimously the designated party line, the definition of frustration index given in (S5) can be specialized to a party-wise (or cluster-wise) frustration index, consisting of the minimum of all the energy functionals given by (S6) over all block diagonal signature matrices S:

$$\zeta = \min_{\substack{S=\text{diag}\{S_1,\ldots,S_{n_\text{p}}\} \\ S_i=s_i\cdot I_{c_i},\ s_i=\pm 1}} e(S) \tag{S7}$$

where c_i, $i = 1, \ldots, n_\text{p}$, is the number of seats gained by the party p_i at the election. The difference with (S5) is that now the signature matrix S is a block diagonal matrix (with n_p blocks): only the relationships between different parties, and not the ones between single MPs, are taken into account. Computing (S7) is much faster than computing (S5). In fact, all energy levels of our parliamentary networks can be exhaustively explored in a systematic way. The resulting energy landscape can be represented as histograms, having in the leftmost point the ground state energy. See Supplementary Fig. 3 for the 29 countries of Supplementary Table 1 in one of the scenarios discussed in the paper (scenario **I**).

S1.3 Dynamical model of decision-making in presence of frustration

Let $\mathcal{G} = (\mathcal{V}, \mathcal{E}, A)$ be a signed network whose node set $\mathcal{V}$ represents a community of agents. To represent a process of decision-making for these agents, we consider the following nonlinear interconnected dynamical model, previously used in (*17–19*),

$$\dot{x} = -\Delta x + \pi A \psi(x), \quad x \in \mathbb{R}^n, \tag{S8}$$

where the state vector $x \in \mathbb{R}^n$ represents the decisions of the agents and $A \in \mathbb{R}^{n\times n}$ is the adjacency matrix of the network $\mathcal{G}$. Each element of the diagonal matrix $\Delta = \{\delta_1, \ldots, \delta_n\}$ is given by $\delta_i = \sum_{i=1}^{n} |a_{ij}|$, $i = 1, \ldots, n$, while π is a scalar positive parameter representing the "social effort" or strength of the commitment among the agents. $\psi(x) = [\psi_1(x_1) \ldots \psi_n(x_n)]^T$ are sigmoidal nonlinear functions. We assume that each function $\psi_i(x_i) : \mathbb{R} \to \mathbb{R}$ is monotone, i.e., $\frac{\partial \psi_i}{\partial x_i}(x_i) > 0 \; \forall x_i \in \mathbb{R}$, with unit slope at the origin, $\frac{\partial \psi_i}{\partial x_i}(0) = 1$, and saturated behavior: $\lim_{x_i \to \pm\infty} \psi_i(x_i) = \pm 1$. From previous works, such as (*16, 19*), we know that the existence of equilibrium points is determined by the social effort parameter π: when $\pi < \pi_1$ the origin is the unique (and globally asymptotically stable) equilibrium point of the system (S8). When $\pi = \pi_1$ the system (S8) undergoes a pitchfork bifurcation (i.e., the number of equilibria "jumps" from one to three) and for $\pi > \pi_1$ two alternative equilibrium points x^* and $-x^*$ appear, which are locally asymptotically stable, while the origin becomes unstable. Finally, when $\pi = \pi_2$ the system (S8) undergoes a second pitchfork bifurcation and for $\pi > \pi_2$ it admits multiple equilibria. In the context of social networks, where each equilibrium point corresponds to a possible decision made by the agents, the behavior of the system can be interpreted as follows: when $\pi < \pi_1$ the social effort of the agents is not enough to reach a nontrivial decision. When π grows past the first threshold value π_1, the higher strength of commitment among the agents leads to two possible alternative decisions. Finally, when $\pi > \pi_2$, the "overcommitment" of the agents leads to a situation in which multiple decisions are possible. We are interested in the case when π belongs to the interval $[0, \pi_2]$, since the agents have to choose among no nontrivial decision (in $[0, \pi_1]$) and among two alternative nontrivial decisions (in $[\pi_1, \pi_2]$).

The threshold values π_1 and π_2 are functions of the two smallest eigenvalues of the normalized signed Laplacian of the network, $\mathcal{L} = I - \Delta^{-1}A$: $\pi_1 = \frac{1}{1-\lambda_1(\mathcal{L})}$ and $\pi_2 = \frac{1}{1-\lambda_2(\mathcal{L})}$. Moreover, at $\pi = \pi_1$ the nontrivial equilibria $\pm x^*$ appear on span$\{v_1(\mathcal{L})\}$, where $v_1(\mathcal{L})$ is the eigenvector relative to $\lambda_1(\mathcal{L})$, see (*16*). The values of $\lambda_1(\mathcal{L})$ and $\lambda_2(\mathcal{L})$ depend not only on the network structure but also on its *signature*: as explained in Section S1.2, $\lambda_1(\mathcal{L}) = 0$, and consequently π_1 is fixed and constant to $\pi_1 = 1$, if and only if the network $\mathcal{G}$ is structurally balanced. When this is not the case, $\lambda_1(\mathcal{L})$ grows with the frustration of the network, implying that also π_1 increases. This means that networks which are structurally unbalanced need a higher social effort (π_1) from the agents in order to converge to a nontrivial decision. Regarding π_2, its value depends on $\lambda_2(\mathcal{L})$, which is the algebraic connectivity of the network in the structurally balanced case. If the network is structurally unbalanced, unlike $\lambda_1(\mathcal{L})$, $\lambda_2(\mathcal{L})$ remains nearly independent from the frustration of the network, i.e., even if ϵ grows, $\lambda_2(\mathcal{L})$ remains almost constant. This means that the interval $[\pi_1, \pi_2]$ in which nontrivial decisions appear for the social effort parameter π shrinks as the frustration of the network ϵ increases. These results, shown in (*16*), can be summarized as follows:

$$\pi_1 = \frac{1}{1-\lambda_1(\mathcal{L})} \begin{cases} = 1 \text{ fixed}, & \text{if } \mathcal{G} \text{ is structurally balanced} \\ \text{grows with the frustration } \epsilon, & \text{if } \mathcal{G} \text{ is structurally unbalanced} \end{cases} \tag{S9a}$$

$$\pi_2 = \frac{1}{1-\lambda_2(\mathcal{L})} \begin{cases} \text{depends on the algebraic connectivity of } \mathcal{G}, & \text{if } \mathcal{G} \text{ is structurally balanced} \\ \text{independent from the frustration } \epsilon, & \text{if } \mathcal{G} \text{ is structurally unbalanced} \end{cases} \tag{S9b}$$

As introduced in Section S1.1, each network $\mathcal{G}_{\text{country, date}}$ considered in this work is composed of n_p clusters, where n_p is the total number of parliamentary political parties. Nonetheless, we still expect a behavior similar to the one described in equation (S9) for the first threshold value π_1 also for the party-wise frustration of the network, whose definition was given in (S7).

This can be shown with a numerical example, where we consider a network $\mathcal{G} = (\mathcal{V}, \mathcal{E}, A)$ with $n = \text{card}\,(\mathcal{V}) = 500$ individuals and n_p clusters, representing an all-against-all scenario (scenario **I**), whose adjacency matrix A is hence a $n_\text{p} \times n_\text{p}$ block matrix described by (S1), where $w_{ii} = 1$ for all $i = 1, \ldots, n_\text{p}$ and $w_{ij} = -1$ for all $i, j = 1, \ldots, n_\text{p}$ and $j \neq i$. We decided to vary the number of parties and, for each

n_p, to consider 1000 (unique) randomly selected vectors $[c_1, \dots, c_{n_\mathrm{p}}]$ s.t. $c_i \in [1, n]$ and $\sum_{i=1}^{n_\mathrm{p}} c_i = n$, where c_i is the size of each party, $i = 1, \dots, n_\mathrm{p}$. Supplementary Figure 4 shows the results for $n_\mathrm{p} = 3, 4, \dots, 20$. Supplementary Figure 4A illustrates how factors such as the number of political parties and the size of each party i (c_i) influence the frustration: an increase in the number of parties (n_p, left panel) or a decrease in the maximum number of MPs per party ($\max_i c_i$, right panel) both lead to an increase in the frustration in average, although the variance is extremely large. Instead, Supplementary Fig. 4B shows that as the frustration of the network increases so does the threshold value π_1. As a consequence, a higher social effort will be required from the agents to achieve a nontrivial decision.

S2 Application: from parliamentary networks to government formation

For a given country and general election, we aim to use our parliamentary networks to predict:

1. The duration of the negotiation phase that leads to the formation of a post-electoral cabinet.
2. The composition of the governmental coalition that sustains such a successful post-electoral cabinet.

In both cases we are interested only in the cabinet that is being formed immediately after a general election. Inter-election government formation processes are often following different rules, see (*20, 21*).

S2.1 Frustration vs government negotiation days

We are interested in the possible correlation between the frustration of each network $\mathcal{G}_{\text{country, date}}$, measured by ζ (formula (S7)), and the numbers of days between the election date and the date the government is sworn in. The *rationale* behind this is that when no clear majority has emerged from the electoral ballot, there is uncertainty in the composition of the candidate cabinet, and the political parties require more time in order to overcome their differences and tensions if they want to establish coalitions which can ensure a majority in the Parliament. Mathematically, the link between the two properties is given by the dynamical model described in Section S1.3. Lack of a clear electoral winner corresponds to a parliamentary network with high frustration index ζ.

In our model (S8), a high frustration raises the value of the bifurcation point π_1, meaning that achieving a nontrivial decision (i.e., giving a confidence vote to a government) requires a high "social effort", here interpreted as duration of the negotiation process among the parties. A graphical representation can be seen in Fig. 1C of the paper.

To check if this hypothesis is valid for our data, we compute the Pearson correlation (r) between ζ and the number of days between the general election and the date the government is sworn in. The higher the value for r (which ranges between -1 and 1), the "stronger" the evidence that frustration indeed influences the dynamics of the government formation process. The resulting values are shown in Fig. 2A of the main text.

S2.2 Minimum energy government coalition

For each parliamentary network $\mathcal{G}_{\text{country, date}} = (\mathcal{V}, \mathcal{E}, A)$, the energy landscape is obtained by computing the energies in all the 2^{n_p} possible block signature matrices $S = \text{diag}\{S_1, \dots, S_{n_\mathrm{p}}\}$, with $S_i = s_i \cdot I_{c_i}$ and

$s_i = \pm 1 \, \forall \, i$. The party-wise frustration index is the minimum of such energies, as computed in (S7). Denote

$$\begin{aligned} S_{\text{best}} &= \arg\min_{S} e(S) \\ &\text{s.t. } (i) \; S = \text{diag}\{s_1 \cdot I_{c_1}, \dots, s_{n_\text{p}} \cdot I_{c_{n_\text{p}}}\}, \; s_i = \pm 1, \\ &\quad (ii) \sum_{i:\, s_i = +1} c_i \geq \sum_{i:\, s_i = -1} c_i. \end{aligned} \tag{S10}$$

the block diagonal signature matrix (with more diagonal elements equal to $+1$ than -1) which gives the minimum of the energy functional, i.e., $\zeta = e(S_{\text{best}})$. Consequently, $-S_{\text{best}}$ is a block diagonal signature matrix with more diagonal elements equal to -1 than $+1$; observe that $e(-S_{\text{best}}) = e(S_{\text{best}}) = \zeta$. In the paper, S_{best} is associated with "success" of a confidence vote to form a government cabinet, and $-S_{\text{best}}$ to "failure". Such ground state can in general be degenerate (that is, several pairs $\pm S_{\text{best}}$ may give the same frustration index ζ).

Let $S_{\text{best}} = \text{diag}\{s_{1,\text{best}} \cdot I_{c_1}, \dots, s_{n_\text{p},\text{best}} \cdot I_{c_{n_\text{p}}}\}$. The two party sets $\{p_i : \; s_{i,\text{best}} = +1\}$ and $\{p_i : \; s_{i,\text{best}} = -1\}$, where p_i is the i-th party, form a minimum energy partition of the set $\mathcal{P} = \{p_i, \, i = 1, \dots, n_\text{p}\}$ corresponding to (the positive and negative diagonal elements of) the block diagonal signature matrix S_{best}.

For the case of "success" (the vast majority of our data), the intuition is that the set $\mathcal{P}_{\text{best}} := \{p_i \in \mathcal{P} : \; s_{i,\text{best}} = +1\}$, which corresponds to a majority coalition of parties, should contain the possible new government coalition, plus the parties that support it in parliament without officially participating to it. In other words, $\mathcal{P}_{\text{best}}$ should be a superset of the set $\mathcal{P}_{\text{gov}} = \{p_i \in \mathcal{P} : \; p_i \in \text{gov}\}$, i.e., the post-electoral cabinet that actually took place.

Let $S_{\text{gov}} = \text{diag}\{s_{1,\text{gov}} \cdot I_{c_1}, \dots, s_{n_\text{p},\text{gov}} \cdot I_{c_{n_\text{p}}}\}$ be a block diagonal signature matrix whose elements $s_{i,\text{gov}}$, $i = 1, \dots, n_\text{p}$, are defined as follows,

$$s_{i,\text{gov}} = \begin{cases} +1, & p_i \in \mathcal{P}_{\text{gov}} \\ -1, & p_i \notin \mathcal{P}_{\text{gov}}. \end{cases}$$

To evaluate how our predictions (S_{best}) reflect the actually formed government, we introduce the following indexes:

$$\rho_{\text{gov}} = \frac{\text{card}\,(\mathcal{P}_{\text{best}} \cap \mathcal{P}_{\text{gov}})}{\text{card}\,(\mathcal{P}_{\text{gov}})} \tag{S11}$$

$$\eta_{\text{gov}} = 1 - \frac{e(S_{\text{gov}}) - \zeta}{\max_S e(S) - \zeta}. \tag{S12}$$

The closer the value of ρ_{gov} is to 1, the better our estimate represents the actual cabinet composition. η_{gov} represents instead how close "energetically" our guess (i.e., ζ) is to the true government energy $e(S_{\text{gov}})$. In (S12), $e(S)$ and ζ are given by (S6) and (S7) respectively, $e(S_{\text{gov}})$ is the energy in correspondence of S_{gov}, and S in $e(S)$ is used as in (S7) to indicate a signature matrix with n_p blocks, i.e., $S = \text{diag}\{s_1 \cdot I_{c_1}, \dots, s_{n_\text{p}} \cdot I_{c_{n_\text{p}}}\}$, $s_i = \pm 1 \, \forall \, i$.

As already mentioned, several $\pm S_{\text{best}}$ may give the same frustration index ζ (i.e., the ground state is degenerate). Moreover, among those, there might exist signature matrices S_{best} determining party sets $\{p_i \in \mathcal{P} : \; s_{i,\text{best}} = +1\}$ and $\{p_i \in \mathcal{P} : \; s_{i,\text{best}} = -1\}$ with equal total number of seats, $\sum_{i:\, s_{i,\text{best}} = +1} c_i = \sum_{i:\, s_{i,\text{best}} = -1} c_i = \frac{n}{2}$ (equality holds in (S10)(ii)). We consider the latter as inconclusive cases, meaning that our analysis is not able to identify the possible government. More specifically, these issues are handled as follows.

(i) In presence of multiple S_{best} and multiple valid values for ρ_{gov}, we consider the signature matrix S_{best} giving maximum possible value for ρ_{gov}.

(ii) In presence of multiple S_{best} all representing inconclusive cases for the analysis, we define $S'_{\text{best}} = \text{diag}\{s'_{1,\text{best}} \cdot I_{c_1}, \ldots, s'_{n_\text{p},\text{best}} \cdot I_{c_{n_\text{p}}}\}$ as the signature matrix giving minimum possible energy while satisfying a "non-inconclusive" condition, $\sum_{i:\, s'_{i,\text{best}}=+1} c_i > \sum_{i:\, s'_{i,\text{best}}=-1} c_i$, that is

$$
\begin{aligned}
S'_{\text{best}} = \arg\min_{S} \; & e(S) \\
\text{s.t.} \; & (i) \; S = \text{diag}\{s_1 I_{c_1}, \ldots, s_{n_\text{p}} I_{c_{n_\text{p}}}\}, \; s_i = \pm 1; \\
& (ii) \sum_{i:\, s_i=+1} c_i > \sum_{i:\, s_i=-1} c_i.
\end{aligned}
\tag{S13}
$$

Then, $\mathcal{P}_{\text{best}} := \{p_i \in \mathcal{P} : \; s'_{i,\text{best}} = +1\}$. If S'_{best} is degenerate, we follow point (i).

Supplementary Fig. 8 illustrates how often degenerate or inconclusive cases occur in scenario **I**.

S2.3 Frustration vs smallest eigenvalue of the normalized signed Laplacian

Our analysis is based on two observations. The first, already mentioned in Sect. S1.3, is a well-studied fact from the literature (*12, 13, 16*), namely that the smallest eigenvalue of the normalized signed Laplacian ($\lambda_1(\mathcal{L})$) grows linearly with the frustration of the network (ζ). The second is that there is a high overlap between the signature of the eigenvector relative to $\lambda_1(\mathcal{L})$ ($v_1(\mathcal{L})$) and the signature matrix corresponding to the ground state, which we denote $S_{\text{best}} = \text{diag}\{s_1 I_{c_1}, \ldots, s_{n_\text{p}} I_{c_{n_\text{p}}}\}$, $s_i = \pm 1$ (S10). Notice that since the adjacency matrix of a parliamentary network is a block matrix, see (S1), then $v_1(\mathcal{L})$ is also a block vector, $v_1(\mathcal{L}) = [v_{1,1} \mathbb{1}^T_{c_1} \; v_{1,2} \mathbb{1}^T_{c_2} \; \ldots \; v_{1,n_\text{p}} \mathbb{1}^T_{c_{n_\text{p}}}]^T$.

To check if the first hypothesis holds for our data, we calculate the Pearson correlation between the smallest eigenvalue of the normalized signed Laplacian, $\lambda_1(\mathcal{L})$, and the frustration of the parliamentary networks, ζ. To check if the second assumption is satisfied, we calculate the overlap between $\pm S_{\text{best}}$ and the signature of $v_1(\mathcal{L})$, defined as

$$
\begin{aligned}
\%\text{ overlap} &= \max\left(1 - \frac{||\text{sign}\,(v_1(\mathcal{L})) - S_{\text{best}}||_1}{n}, 1 - \frac{||\text{sign}\,(v_1(\mathcal{L})) + S_{\text{best}}||_1}{n}\right) \cdot 100 \\
&= \max\left(1 - \frac{\sum_{i=1}^{n_\text{p}} c_i |\text{sign}\,(v_{1,i}) - s_i|}{n}, 1 - \frac{\sum_{i=1}^{n_\text{p}} c_i |\text{sign}\,(v_{1,i}) + s_i|}{n}\right) \cdot 100,
\end{aligned}
\tag{S14}
$$

where $n = \sum_{i=1}^{n_\text{p}} c_i$ is the total number of MPs and $\|\cdot\|_1$ indicates the 1-norm.

The average values of correlation between $\lambda_1(\mathcal{L})$ and the frustration of the parliamentary networks ζ, and the average overlap (S14) between S_{best} and the signature of $v_1(\mathcal{L})$ (the eigenvector related to $\lambda_1(\mathcal{L})$) are reported in Supplementary Table 2 for each scenario (**I**, **II**, **III**).

S2.4 Frustration vs fractionalization index

As shown in previous studies such as (*20, 22*) (and as intuitively clear), parliamentary fragmentation is one of the main factors influencing the duration of the government negotiation process. Such fragmentation is often measured in terms of the Laakso-Taagepera effective number of parties (*23*), or in terms of the strictly related fractionalization index (*24*). In this section we investigate (analytically) how the frustration of the signed parliamentary networks (of scenario **I**) is related to the fractionalization index (which carries the same information of the effective number of parties).

The effective number of parties (denoted N_2) is defined (see (*23*)) as the inverse of the sum of squares of the shares of seats of each party,

$$N_2 = \frac{1}{\sum_{i=1}^{n_\mathrm{p}} \left(\frac{c_i}{n}\right)^2} = \frac{n^2}{\sum_{i=1}^{n_\mathrm{p}} c_i^2}, \tag{S15}$$

where c_i is the size of the i-th party, n_p is the total number of parties and n is the total number of MPs, while the fractionalization index (denoted F) is defined (see (*24*)) as $F = 1 - \frac{1}{N_2}$.

To compare the frustration of the signed networks to these indexes, we first need to rewrite the expression of the weighted energy functional $e(S)$, see (S6), by taking into consideration that the parliamentary networks, as introduced in Section S1.1 and represented by $\mathcal{G}_{\text{country, date}}$, can be partitioned in n_p clusters (corresponding to the political parties), each of size c_i ($i = 1, \dots, n_\mathrm{p}$). Indeed, this means that the adjacency matrix A (see (S1)) and the diagonal matrix $\Delta = \mathrm{diag}\{|A|\mathbb{1}\}$ (introduced in Section S1.2) are block matrices. In particular, $\Delta = \mathrm{diag}\{\delta_1 I_{c_1}, \dots, \delta_{n_\mathrm{p}} I_{c_{n_\mathrm{p}}}\}$ where each block $\delta_i I_{c_i}$ satisfies

$$(\delta_i I_{c_i})\mathbb{1}_{c_i} = \sum_{j\in\mathcal{I}} |A_{ij}|\mathbb{1}_{c_j} = \sum_{\substack{j\in\mathcal{I}\\ j\neq i}} |w_{ij}| E_{c_i c_j}\mathbb{1}_{c_j} + (E_{c_i} - I_{c_i})\mathbb{1}_{c_i} = \Big(\sum_{j\in\mathcal{I}} |w_{ij}| c_j - 1\Big)\mathbb{1}_{c_i}, \quad i \in \mathcal{I} \tag{S16}$$

where $\mathcal{I} = \{1, \dots, n_\mathrm{p}\}$. Then the weighted energy functional $e(S)$, where $S = \mathrm{diag}\{s_1 I_{c_1}, \dots, s_{n_\mathrm{p}} I_{c_{n_\mathrm{p}}}\}$ with $s_i = \pm 1 \;\forall\, i$, can be rewritten as follows:

$$e(S) = \frac{1}{2}\mathbb{1}_n^T \Delta^{-1} \left(|A| - SAS\right) \mathbb{1}_n = \frac{1}{2} \sum_{i,j\in\mathcal{I}} \frac{c_i c_j}{\delta_i} (|w_{ij}| - s_i w_{ij} s_j), \tag{S17}$$

where w_{ij} is the weight between the i-th and j-th party.

In scenario **I** all party-party weights are negative and equal to -1 ($w_{ij} = -1$ for all $j \neq i$), which implies that $\delta_i = n - 1$ for all $i \in \mathcal{I}$ and that (S17) becomes

$$e(S) = \frac{1}{2(n-1)} \sum_{i,j\in\mathcal{I},\, j\neq i} c_i c_j (1 + s_i s_j) = \frac{1}{n-1} \cdot \sum_{\substack{i,j\in\mathcal{I},\, j\neq i\\ s.t.\ s_i s_j > 0}} c_i c_j.$$

Let $\mathcal{C}_+ = \{i \in \mathcal{I} :\ s_i = +1\}$ and $\mathcal{C}_- = \{i \in \mathcal{I} :\ s_i = -1\}$ be the two node subsets defined by a signature matrix $S = \mathrm{diag}\{s_1 I_{c_1}, \dots, s_{n_\mathrm{p}} I_{c_{n_\mathrm{p}}}\}$, and let $n_{\mathcal{C}_+} = \sum_{i\in\mathcal{C}_+} c_i$ and $n_{\mathcal{C}_-} = \sum_{i\in\mathcal{C}_-} c_i$. Notice that $\mathcal{C}_+ \cap \mathcal{C}_- = \emptyset$ and that $n_{\mathcal{C}_+} + n_{\mathcal{C}_-} = n$. Then, the calculation of the frustration of a signed parliamentary network in scenario **I** reduces to

$$\begin{aligned}
\zeta &= \frac{1}{n-1} \cdot \min_{\substack{\mathrm{diag}\{s_1,\dots,s_{n_\mathrm{p}}\}\\ s_i = \pm 1}} \sum_{\substack{i,j\in\mathcal{I},\, j\neq i\\ s.t.\ s_i s_j > 0}} c_i c_j \\
&= \frac{1}{n-1} \cdot \min_{\mathcal{C}_+\subseteq\mathcal{I}} \left(\sum_{i,j\in\mathcal{C}_+,\, j\neq i} c_i c_j + \sum_{i,j\in\mathcal{I}\setminus\mathcal{C}_+,\, j\neq i} c_i c_j \right) \\
&= \frac{1}{n-1} \cdot \left(\min_{\mathcal{C}_+\subseteq\mathcal{I}} \Big(\sum_{i,j\in\mathcal{C}_+} c_i c_j + \sum_{i,j\in\mathcal{I}\setminus\mathcal{C}_+} c_i c_j \Big) - \sum_{i\in\mathcal{I}} c_i^2 \right) \\
&= \frac{1}{n-1} \cdot \left(\min_{\mathcal{C}_+\subseteq\mathcal{I}} \big(n_{\mathcal{C}_+}^2 + (n - n_{\mathcal{C}_+})^2 \big) - \sum_{i\in\mathcal{I}} c_i^2 \right)
\end{aligned}$$

$$= \frac{1}{n-1} \cdot \left(2 \min_{\mathcal{C}_+ \subseteq \mathcal{I}} \left(n_{\mathcal{C}_+}^2 - n \cdot n_{\mathcal{C}_+} \right) + n^2 - \sum_{i \in \mathcal{I}} c_i^2 \right)$$

$$= \frac{n^2}{n-1} \cdot \left(-2 \max_{\mathcal{C}_+ \subseteq \mathcal{I}} \left(\frac{n_{\mathcal{C}_+}}{n} - \frac{n_{\mathcal{C}_+}^2}{n^2} \right) + \underbrace{1 - \frac{\sum_{i \in \mathcal{I}} c_i^2}{n^2}}_{=1-\frac{1}{N_2}=F} \right). \tag{S18}$$

Equation (S18) shows that the frustration of the signed networks of scenario **I** is proportional to the difference between the fractionalization index F and a term which is related to the size of the minimum winning coalition. To obtain some insight on the frustration of scenario **I** (and in particular on the first term of equation (S18)), let $\mathcal{P}_{\text{best}}$ be the solution of the minimization - or, maximization, depending on the sign - problem in (S18) (see also Section S2.2) and $n_{\mathcal{P}_{\text{best}}} = \frac{n}{2} + E_{\text{best}}$ where $E_{\text{best}} \in [0, \frac{n}{2}]$ is the *number of seats in excess* (with respect to 50% of the total number of seats). Then the frustration (up to a constant multiplicative term) can be written as:

$$\zeta \cdot \frac{n-1}{n^2} = \underbrace{F}_{\text{fractionalization index}} - \frac{1}{2} + \underbrace{\frac{1}{2} \left(\frac{E_{\text{best}}}{n/2} \right)^2}_{\text{“distance” of } \mathcal{P}_{\text{best}} \text{ from } 50\%} . \tag{S19}$$

In conclusion, equation (S19) shows that the frustration of the signed parliamentary networks of scenario **I** is proportional to the frustration index F and to the “distance” of the the majority $\mathcal{P}_{\text{best}}$ (in correspondence of minimum winning coalition S_{best}) to 50% of the total number of seats. Supplementary Figure 9 shows the linear regression plots between the fractionalization index and the frustration of the parliamentary networks for each country and election. The average value of correlation is 0.99 (see Fig. 2D in the main manuscript). Moreover, Supplementary Fig. 9 shows that countries with a lower value of correlation (such as Albania, North Macedonia, Moldova, UK) are characterized by minimum winning coalitions S_{best} with higher excess of seats E_{best}.

S2.5 Description of the results

A summary of the results obtained for all the scenarios mentioned in Fig. 1A of the paper, in terms of correlation between duration of government negotiations and frustration, indexes evaluating how good our estimates of the cabinet composition are, and correlation between fractionalization index and frustration is given in Fig. 2 in the main text.

For scenario **I**, the number of government negotiation days and the frustration for each country are shown in Supplementary Fig. 2 panel A and B, respectively. The percentages of correct government predictions, in terms of ρ_{gov} and η_{gov}, are given in Supplementary Fig. 2C and S2D, respectively. Supplementary Fig. 3 shows the energy functionals for each country and election year; the red line represents the energy corresponding to the government coalition, $e(S_{\text{gov}})$.

Regression plots (see Section S2.6 for more details on the linear regression analysis) between the government negotiation days and the frustration of the parliamentary networks with the corresponding values of correlation are shown in Fig. 3 (in the main text), Supplementary Fig. 5 and Supplementary Fig. 6, respectively for scenario **I**, **II** and **III**.

The correlation r_{country} computed for each country and scenario, between the duration of coalition negotiations and the party-wise frustration of the network, is given in Fig. 2A in the main text: overall, the values of correlation for all scenarios are positive and above 0.4 with few exceptions which often admit a clear explanation and will be discussed in Section S2.7. In scenario **III** we consider 10000 different values for the political positions (fixed in time: the same for all elections) in the left-right scale, as explained in Section S1.1: for each country, the “optimal” choice of positions is identified as the one giving the best

value of correlation, depicted in Supplementary Fig. 1C with a blue circle. This value is then used in Fig. 2 (scenario **III**) and Supplementary Fig. 6. As can be seen in Supplementary Fig. 1C, the positions we assign to the left-right grid significantly affect the correlation, hence the optimal r_{country} may be far from the average of the correlations obtained for each country. This could also explain why the results obtained in scenario **II** are worse, in terms of correlation, than the ones obtained in scenario **I**, where the networks we consider are unweighted.

S2.6 Influential points of the regression are important

Consider the "frustration vs. days" regression plots for a country (depicted in Fig. 3 in the main text, Supplementary Fig. 5 and Supplementary Fig. 6 for scenarios **I**, **II** and **III**), and let x_{date} and y_{date} be, respectively, the party-wise frustration of the network $\mathcal{G}_{\text{country, date}}$ and the duration of coalition negotiations after the election specified by "date". We say that a point $(x_{\text{date}}, y_{\text{date}})$ is an *outlier* for that country if the value of y_{date} does not follow the linear regression line, meaning that this value is unusual given x_{date}. Instead, it is a *high leverage* point if it has "extreme" x_{date} value, i.e., this value is unusual given y_{date}. Finally, we say that the point is *influential* if it influences the slope of the regression line. See (*25, 26*) for a detailed explanation of outliers, high leverage and influential points. Tests such as residuals, leverage and delete-1 statistics are used to identify the possible outliers, high leverage and influential points. In practice, given the linear regression model, outliers are observations whose studentized (also denoted externally studentized or studentized deleted) residuals are greater than 3 in absolute value and whose standardized (or internally studentized) residuals are greater than 2 in absolute value. Observations whose leverage statistics have values greater than $\frac{2p}{N}$, where $p = 2$ is the number of regression coefficients and N is the total number of observations (in this case the number of elections considered for each country), are identified as high leverage. Finally, observations whose Cook's distance is greater than three times the average Cook's distance, and whose Dffits (Difference in fits) values are greater than $2\sqrt{\frac{p}{N}}$, are identified as influential points.

We are particularly interested in observations marked as outliers and as both high leverage and influential, because they often represent unexpected events or difficult situations, in terms of either frustration or government negotiation duration, or both. In the vast majority of cases indeed they correspond to both high frustration and long negotiation times.

S2.7 A brief discussion on national rules and traditions influencing the duration of the government negotiation talks

It is beyond the scope of this paper to make a thorough analysis of the additional factors influencing the duration of coalition negotiations, and we refer the reader to (*27–30*). However, to shed some light on the systematic differences in the government negotiation times across the various countries (see Supplementary Fig. 2A), it is worth mentioning that the countries characterized by "negative" parliamentarism tend to have short cabinet formation processes. We say that a certain country has "negative" parliamentarism if the government (in order to rule) does not need to win a vote of confidence in the Parliament (as in the UK), or if the majority of the parties does not vote against it in the Parliament (as in Sweden), while it has "positive" parliamentarism otherwise (*30*). The presence of minority governments and, most importantly, average short government formation processes is related to countries having "negative" parliamentarism: for instance Denmark, Finland, Iceland, Norway, Sweden and United Kingdom, see (*20, 28, 30*) and Supplementary Fig. 7B.

In other countries the duration of the negotiation period is defined (and limited) by the constitution. It is worth observing that Estonia and Greece, two of the three countries showing a negative correlation between frustration and negotiation days in our scenario **I** and **II**, are among them. In Estonia, according to Article

89 of the Constitution, a limited time is given to the candidate Prime Minister to form a new Government: the President has 14 days to appoint a candidate Prime Minister, who in turn has to win a confidence vote in not more than 24 days. In case of failure the President can nominate (within 7 days) another candidate and the procedure repeats. Again, in case of failure, the Parliament nominates a candidate who has 14 days to win a vote of confidence. For Greece, as Article 37 of the Constitution states, the leader of the party with relative majority receiving from the President of the Republic the task to form a coalition (that has to enjoy the confidence of the Parliament) has 3 days to succeed. In case of failure, the task is given to the leader of the second, and then third, largest party in the Parliament. If all the rounds of government negotiations fail new elections are called.

S2.8 A brief discussion on elections resulting in a hung parliament

It is interesting to describe in some detail some cases of hung parliament mentioned in the main manuscript, whose corresponding data points in Fig. 3 (for scenario **I**, Supplementary Fig. 5 and 6 for scenarios **II** and **III**) are both influential and high leverage points.

The 2006 Czech election saw a perfect partition of the parliament into parties of the left, Czech Social Democratic Party (ČSSD) and Communist Party of Bohemia and Moravia (KSČM) (that together won 100 of the 200 seats), and centre-right parties, Civic Democratic Party (ODS), Green Party (SZ) and Christian and Democratic Union - Czechoslovak People's Party (KSU-ČSL) (that collectively won the other 100 seats) (*31*). In our framework, a parliament split into two identical halves represents a degenerate situation which escapes classification: the frustration can be very low but no majority exists. Indeed in our method the partition mentioned above corresponds to the ground state, i.e., our S_{best} consists of exactly 50% of $+$ and 50% of $-$. The duration of the negotiation process cannot be predicted in degenerate cases like this because the two "real-life" equal size factions are also matching ideological polarization.

The 2016 and 2020 elections in Ireland, unlike the previous elections, resulted in no clear majority (or possible government coalition) and produced instead the most fragmented Dáils in history (which explains the higher levels of frustration): the number of parties in the parliament increased to 12 (from an average of 8 in the previous elections) and, while in the previous elections the percent distance between the first two parties (i.e., difference between their shares of seats in the parliament) had been at least of 19%, in the following elections it decreased, with the two biggest parties differing by as little as 3.2% in 2016 and 2.3% in 2020. However, differently from the 2016 election, in the 2020 election three parties (Fianna Fáil, Sinn Féin, Fine Gael) won roughly the same number of seats, which is reflected in a fractionalization index (or effective number of parties, see (S15)) for the 2020 election higher than the one for the 2016 election: this meant that, for the first time, at least three parties were needed to form a majority coalition (*32*). After the 2016 election, most of the parties did not want to participate in the government coalition and after 70 days of negotiations a "confidence and supply" deal was signed between Fine Gael and Fianna Fáil, with Fianna Fáil abstaining from voting against Fine Gael (*33*), while after the 2020 election, both Fine Gael and Fianna Fáil did not want to collaborate with Sinn Féin and decided instead to form a grand coalition with the Green Party.

In Spain, after the 2015 general election no agreement was reached between the parties before the deadline imposed by the Article 99 of the Spanish constitution (corresponding to two months from the first vote for investiture). A snap election was then called in June 2016 and after 131 days the Rajoy II government, composed by members of the People's Party (PP) and independents, was sworn in. Single-party (minority) governments have always been common in Spain, however these elections saw a change from a nearly two-party system, i.e., majority of votes won by two parties, PP and Spanish Socialist Workers' Party (PSOE), to an effectively multi-party system (*34*) which increased the complexity of the cabinet formation process. Similarly, the government negotiations failed after the April 2019 legislative election, and the King had to

call new elections in November 2019, which gives us a second "failure point" for Spain.

In 2017 in Germany 171 days passed from the election date before the Merkel IV cabinet was sworn in. Neither of the possible coalitions, Christian Democratic Union/Christian Social Union in Bavaria (CDU/CSU) and Free Democratic Party (FDP) or Social Democratic Party (SPD) and The Greens (GRÜNE), obtained a majority of seats in the parliament, while a new party, the far-right Alternative for Germany (AfD), managed to enter the parliament for the first time (*35*). Since none of the parties wanted to collaborate with AfD, a compromise between parties with different ideological views had to be found: after a failed attempt to form a "Jamaica Coalition" (CDU/CSU, GRÜNE and FDP) the new government comprised a grand coalition between CDU/CSU and SPD.

S2.9 A brief discussion on elections where the government negotiations failed

In our analysis of government formation, most of the legislative elections (255 out of 260 elections) are followed by successful cabinet negotiation talks, after which a cabinet is approved by the parliament and sworn in. In three countries, Czech Republic, Greece and Spain, we found however instances of failure of the government negotiation talks, in the form of a failed vote of investiture or expiration of the deadline set by the Constitution.

In the Czech Republic the candidate government needs to pass an investiture vote (of simple majority) within 30 days after its appointment (Article 68 of the Czech Constitution). In 2006, Topolánek (leader of ODS) decided to form a minority government even without managing to secure support from the other political parties (*31*). Topolánek's first cabinet was sworn in on September 4 but lost a confidence vote on October 3, and cabinet negotiations had to restart. The deadlock was broken only after 220 days, thanks to the abstention of some MPs from ČSSD, and the Topolánek II cabinet (centre-right coalition of ODS, SZ and KSU-ČSL) was sworn in (*31*). A similar situation (failure and then success at the same election) occurred again in the 2017 Czech election. In 2017, none of the political parties wanted to cooperate with Babiš due to the criminal fraud charges he was facing (*36*) and the Babiš first cabinet (ANO 2011 only), formed on December 13, failed to pass an investiture vote on January 16. In the cabinet negotiations that followed, ANO 2011 managed to reach a coalition agreement with ČSSD and to obtain external support from KSČM, and after 249 days the Babiš second cabinet was sworn in.

In Greece after the May 2012 legislative election none of the three leaders tasked to form a government managed to negotiate a cabinet coalition and, following the Article 37 of the Greek Constitution (see also Section S2.7), 10 days after the election day a caretaker cabinet was appointed and new elections were announced for June.

In Spain, see Article 99 of the Spanish Constitution, if the candidate government does not succeed in obtaining the confidence of the parliament (i.e., if it loses both a first vote of absolute majority and, 48 hours after, a second vote of simple majority) then a new candidate cabinet can try to obtain the confidence of the parliament. If within two months after the first vote for investiture no cabinet has managed to win a vote of confidence, the King can call new elections. After the 2008, 2016 and November 2019 elections the candidate cabinets managed to win the confidence of the parliament (with a vote of simple majority) while, after the 2015 and April 2019 elections, the government negotiations failed and the King had to call new elections.

For each of these three countries, Supplementary Fig. 7A reports the election dates, the government sworn in dates and the government negotiations failure dates, determined for the Czech Republic as the date the cabinet (which later failed to pass the investiture vote) was formed, for Greece as the date the caretaker cabinet was sworn in, and for Spain as the date corresponding to two months after the first vote for investiture.

Figure 3 in the main text plots the duration of the government negotiation talks (calculated as number

of days between the election date and the sworn in date and denoted *days*, see Supplementary Fig. 7A) vs the frustration of the parliamentary networks, for scenario **I** (scenario **II** and **III** are reported respectively in Supplementary Fig. 5 and Supplementary Fig. 6). For Czech Republic, Greece and Spain, the blue data points (and corresponding regression line and value of correlation) correspond to elections followed by successful votes of investiture (the May 2012 Greek election and the 2015 and April 2019 Spanish elections are hence excluded), while the yellow data points (and corresponding regression line and value of correlation between parentheses) include all the elections: in this case the variable *days* indicates the number of days between the election date and the government negotiations failure date (see Supplementary Fig. 7A).

S2.10 Analysis of the type of governments formed after the elections

We distinguish between four types of governments (two being minority governments, two majority) that may be formed after a legislative election: first, we consider minority governments where the cabinet coalition has a strict minority in the parliament; second, minority governments whose coalition of parties holds exactly half of the seats in the parliament; third, majority governments which are minimal winning coalitions; and last, majority governments which are surplus coalitions. A coalition of parties is minimal winning if removing a party implies loss of majority in the parliament (*37*), and it is denoted surplus otherwise.

Supplementary Fig 7B shows the type of government the countries we have considered in our analysis have formed: of 257 analyzed governments, 61 (23.74%) are minority, 5 (1.95%) are cabinet coalitions holding exactly 50% of the seats in the parliament, 131 (50.97%) are minimal winning, and 60 (23.35%) are surplus coalitions.

In Scenario **I**, computing the frustration is equivalent to find the minimum winning coalition (represented by the group of parties achieving majority in S_{best}), that is, a coalition of parties that is minimal winning and also minimizing the functional $e(S)$, meaning that between all possible minimal winning coalitions it is the one with the lowest amount of total MPs. The expectation that governments should be minimal winning is standard in the literature (*20, 37*), even if it has been recently observed that, in reality, it is not uncommon for minority or surplus governments to form (*38, 39*).

S2.11 Analysis of the Italian bicameral parliamentary system

Under the bicameral system of Italy the candidate cabinet needs to win the confidence of both the Chamber of Deputies and the Senate of the Republic (*40*). In what follows we extend the results obtained for the Chamber of Deputies by considering also the network described by the parties winning seats in the Senate of the Republic.

For each election let $\mathcal{G}_\text{C}$ and $\mathcal{G}_\text{S}$ indicate the signed networks of the lower and upper chamber, respectively, and let the party set be defined as $\mathcal{P} = \mathcal{P}_\text{C} \cup \mathcal{P}_\text{S}$, where $\mathcal{P}_\text{C} = \{p_i : p_i \in \text{Chamber of Deputies}\}$ and $\mathcal{P}_\text{S} = \{p_i : p_i \in \text{Senate of the Republic}\}$. For each subset $\mathcal{P}(S)$ of $\mathcal{P}$ described by the party configuration $S = \text{diag}\{s_1, \dots, s_{\text{card}(\mathcal{P})}\}$, where $s_i = 1$ if the party p_i belongs to the subset $\mathcal{P}(S)$ or $s_i = -1$ otherwise, we define the energy of the configuration S as the couple $[e_\text{C}(S_\text{C}), e_\text{S}(S_\text{S})]$, where S_C (S_S) and $e_\text{C}(S_\text{C})$ (resp. $e_\text{S}(S_\text{S})$) indicate the corresponding party configuration and energy functional (S6) of the network $\mathcal{G}_\text{C}$ (resp. $\mathcal{G}_\text{S}$), that is

$$S_\text{X} = \text{diag}\{s_1 \cdot I_{c_1}, \dots, s_{\text{card}(\mathcal{P}_\text{X})} \cdot I_{c_{\text{card}(\mathcal{P}_\text{X})}}\},\ s_i = \begin{cases} +1, & p_i \in \mathcal{P}_\text{X} \cap \mathcal{P}(S) \\ -1, & p_i \in \mathcal{P}_\text{X} \setminus (\mathcal{P}_\text{X} \cap \mathcal{P}(S)) \end{cases},\ \text{X} \in \{\text{C,S}\},$$

where c_i is the number of seats won by party p_i (correspondingly in the Chamber or the Senate), and

$$e_\text{X}(S_\text{X}) = \frac{1}{2} \sum_{i,j \neq i} [|\mathcal{L}(\mathcal{G}_\text{X})| + S_\text{X} \mathcal{L}(\mathcal{G}_\text{X}) S_\text{X}]_{ij}, \quad \text{X} \in \{\text{C,S}\},$$

where $\mathcal{L}(\mathcal{G}_\text{X})$ indicates the normalized signed Laplacian of the network $\mathcal{G}_\text{X}$. We say that a party configuration S is a majority configuration if its parties hold the majority of seats in both chambers of the parliament, that is, if $\sum_{i:\, p_i \in \mathcal{P}_\text{C} \cap \mathcal{P}(S)} c_i > \frac{630}{2}$ and $\sum_{i:\, p_i \in \mathcal{P}_\text{S} \cap \mathcal{P}(S)} c_i > \frac{315}{2}$, where 630 and 315 are the total number of seats in the Chamber and Senate, respectively.

For each election, the energy of all configurations S is depicted in Fig. 6 in the main text, which shows the values $e_\text{S}(S_\text{S})$ vs $e_\text{C}(S_\text{C})$.

Supplementary References

1. K. Strøm, J. Y. Leipart, Policy, Institutions, and Coalition Avoidance: Norwegian Governments, 1945–1990. *American Political Science Review* **87**, 870–887 (1993).

2. E. H. Allern, Survival of a Majority Coalition: The Norwegian Parliamentary Election of 14 September 2009. *West European Politics* **33**, 904–912 (2010).

3. E. H. Allern, R. Karlsen, A Turn to the Right: The Norwegian Parliamentary Election of September 2013. *West European Politics* **37**, 653–663 (2014).

4. E. H. Allern, N. Aylott, Overcoming the fear of commitment: Pre-electoral coalitions in Norway and Sweden. *Acta Politica* **44**, 259–285 (2009).

5. N. Aylott, N. Bolin, Polarising Pluralism: The Swedish Parliamentary Election of September 2014. *West European Politics* **38**, 730–740 (2015).

6. M. Debus, Pre-electoral commitments and government formation. *Public Choice* **138**, 45–64 (2009).

7. I. Inter-Parliamentary Union, New Parline: the IPU's Open Data Platform (2019).

8. M. J. Laver, I. Budge, eds., *Party Policy and Government Coalitions* (Palgrave Macmillan UK, London, 1992).

9. A. Volkens, T. Burst, W. Krause, P. Lehmann, T. Matthieß, N. Merz, S. Regel, B. Weßels, L. Zehnter, The Manifesto Data Collection. Manifesto Project (MRG/CMP/MARPOR). Version 2020b (2020).

10. G. Facchetti, G. Iacono, C. Altafini, Computing global structural balance in large-scale signed social networks. *Proceedings of the National Academy of Sciences* **108**, 20953–20958 (2011).

11. C. Altafini, Consensus Problems on Networks With Antagonistic Interactions. *IEEE Transactions on Automatic Control* **58**, 935–946 (2013).

12. J. Kunegis, S. Schmidt, A. Lommatzsch, J. Lerner, E. W. De Luca, S. Albayrak, *Proceedings of the 2010 SIAM International Conference on Data Mining* (Society for Industrial and Applied Mathematics, Philadelphia, PA, 2010), pp. 559–570.

13. S. Aref, M. C. Wilson, Measuring partial balance in signed networks. *Journal of Complex Networks* **6**, 566–595 (2018).

14. S. Aref, A. J. Mason, M. C. Wilson, An exact method for computing the frustration index in signed networks using binary programming. *arXiv:1611.09030v2 [cs.SI]* (2016).

15. S. Aref, M. C. Wilson, Balance and frustration in signed networks. *Journal of Complex Networks* **7**, 163–189 (2019).

16. A. Fontan, C. Altafini, *2018 IEEE Conference on Decision and Control (CDC)* (IEEE, Miami Beach, FL, USA, 2018), pp. 109–114.

17. P. U. Abara, F. Ticozzi, C. Altafini, Spectral Conditions for Stability and Stabilization of Positive Equilibria for a Class of Nonlinear Cooperative Systems. *IEEE Transactions on Automatic Control* **63**, 402–417 (2018).

18. R. Gray, A. Franci, V. Srivastava, N. E. Leonard, Multiagent Decision-Making Dynamics Inspired by Honeybees. *IEEE Transactions on Control of Network Systems* **5**, 793–806 (2018).

19. A. Fontan, C. Altafini, Multiequilibria Analysis for a Class of Collective Decision-Making Networked Systems. *IEEE Transactions on Control of Network Systems* **5**, 1931–1940 (2018).

20. S. N. Golder, Bargaining Delays in the Government Formation Process. *Comparative Political Studies* **43**, 3–32 (2010).

21. A. Ecker, T. M. Meyer, The duration of government formation processes in Europe. *Research and Politics* **2**, 1–9 (2015).

22. D. Diermeier, H. Eraslan, A. Merlo, A Structural Model of Government Formation. *Econometrica* **71**, 27–70 (2003).

23. M. Laakso, R. Taagepera, The "Effective" number of parties: a measure with application to West Europe. *Comparative Political Studies* **12**, 3–27 (1979).

24. D. W. Rae, M. Taylor, *The Analysis of Political Cleavages* (Yale University Press, 1970).

25. G. James, D. Witten, T. Hastie, R. Tibshirani, *An Introduction to Statistical Learning*, Springer Texts in Statistics (Springer, 2013).

26. M. H. Kutner, C. J. Nachtsheim, J. Neter, W. Li, *Applied Linear Statistical Models Fifth Edition* (McGraw-Hill Irwin, 2005), fifth edn.

27. L. W. Martin, G. Vanberg, Wasting Time? The Impact of Ideology and Size on Delay in Coalition Formation. *British Journal of Political Science* **33**, 323–332 (2003).

28. A. Ecker, T. M. Meyer, Coalition Bargaining Duration in Multiparty Democracies. *British Journal of Political Science* pp. 1–20 (2017).

29. B. E. Rasch, S. Martin, J. A. Cheibub, eds., *Parliaments and Government Formation* (Oxford University Press, 2015).

30. T. Bergman, Constitutional rules and party goals in coalition formation: an analysis of winning minority governments in Sweden, Phd dissertation, Umeå universitet (1995).

31. L. LINEK, Czech Republic. *European Journal of Political Research* **46**, 929–937 (2007).

32. C. Little, Change gradually, then all at once: the general election of February 2020 in the Republic of Ireland. *West European Politics* **44**, 714–723 (2021).

33. E. O'Malley, *How Ireland Voted 2016: The Election that Nobody Won*, M. Gallaghe, M. Marsh, eds. (Springer International Publishing, Cham, 2016), pp. 255–276.

34. T. D. Lancaster, The Spanish general elections of 2015 and 2016: a new stage in democratic politics? *West European Politics* **40**, 919–937 (2017).

35. T. Bräuninger, M. Debus, J. Müller, C. Stecker, Party Competition and Government Formation in Germany: Business as Usual or New Patterns? *German Politics* **28**, 80–100 (2019).

36. S. Hanley, M. A. Vachudova, Understanding the illiberal turn: democratic backsliding in the Czech Republic. *East European Politics* **34**, 276–296 (2018).

37. W. H. Riker, *The Theory of Political Coalitions* (New Haven: Yale University Press, 1962).

38. A. Bassi, Policy preferences in coalition formation and the stability of minority and surplus governments. *Journal of Politics* **79**, 250–268 (2017).

39. M. Golder, S. N. Golder, D. A. Siegel, Modeling the institutional foundation of parliamentary government formation. *Journal of Politics* **74**, 427–445 (2012).

40. F. Russo, *Parliaments and Government Formation* (Oxford University Press, 2015), pp. 136–152.

41. J. Nousiainen, From semi-presidentialism to parliamentary government: Political and constitutional developments in Finland. *Scandinavian Political Studies* **24**, 95–109 (2001).

Country	Structure of Parliament	Election Dates	Number of Seats (n)	Relevant Events
Albania	U	1992, 1996, 1997, 2001, 2005, 2009, 2013, 2017.	140<br>155 (1997 only)	1991 Dissolution of the Social Republic, start of the 4th Republic.
Andorra	U	1993, 1997, 2001, 2005, 2009, 2011, 2015, 2019.	28	1993 Adoption of a new Constitution.
Austria	B	1979, 1983, 1986, 1990, 1994, 1995, 1999, 2002, 2006, 2008, 2013, 2017, 2019	183	–
Belgium	B	1995, 1999, 2003, 2007, 2010, 2014, 2019	150	1993 Belgian Constitution, Belgium becomes a federal state.
Bosnia and Herzegovina	B	1996, 1998, 2000, 2002, 2006, 2010, 2014, 2018	42	1992 Independence from SFR Yugoslavia.<br>1995 New Constitution.
Bulgaria	U	1991, 1994, 1997, 2001, 2005, 2009, 2013, 2014, 2017	240	1991 New Constitution.
Croatia	U	1992, 1995, 2000, 2003, 2007, 2011, 2015, 2016, 2020	140 +M+D[(a)] (from 2000)<br>127 (1995)<br>138 (1992)	1991 Independence from SFR Yugoslavia.
Czech Republic	B	1992, 1996, 1998, 2002, 2006, 2010, 2013, 2017	200	1993 Independence from Czechoslovakia.
Denmark	U	1981, 1984, 1987, 1988, 1990, 1994, 1998, 2001, 2005, 2007, 2011, 2015, 2019	179	–
Estonia	U	1992, 1995, 1999, 2003, 2007, 2011, 2015, 2019	101	1991 Independence from the Soviet Union.
Finland	U	1991, 1995, 1999, 2003, 2007, 2011, 2015, 2019	200	1991 Constitution amendments: the powers of the President are diminished.<br>2000 New constitution. From semi-presidential to parliamentary republic (*41*).
Germany	B	1990, 1994, 1998, 2002, 2005, 2009, 2013, 2017	598+O+L[(b)]	1990 German reunification.
Greece	U	1990, 1993, 1996, 2000, 2004, 2007, 2009, May 2012, Jun 2012, Jan 2015, Sept 2015, 2019	300	–
Hungary	U	1990, 1994, 1998, 2002, 2006, 2010 [(c)]	386	1989 Third Republic.
Iceland	U	1995, 1999, 2003, 2007, 2009, 2013, 2016, 2017	63	1991 From Unicameral to Bicameral Parliament.
Ireland	B	1992, 1997, 2002, 2007, 2011, 2016, 2020	159 (2020)<br>157 (2016)<br>166 (until 2011)	–
Italy	B	1992, 1994, 1996, 2001, 2006, 2008, 2013, 2018	630	1993 New electoral system.
Latvia	U	1993, 1995, 1998, 2002, 2006, 2010, 2011, 2014, 2018	100	1991 Independence from the Soviet Union.
Luxembourg	U	1984, 1989, 1994, 1999, 2004, 2009, 2013, 2018	60 (from 1989)<br>64 (1984)	–
North Macedonia	U	1990, 1994, 1998, 2002, 2006, 2008, 2011, 2014, 2016, 2020	120 + A[(d)]	1991 Independence from SFR Yugoslavia (officially recognized in 1993).

Continued on next page

Table S1 – continued from previous page

Moldova	U	1994, 1998, 2001, 2005, July 2009, 2010, 2014, 2019 [(e)]	101 (from 1998)<br>104 (1994)	1991	Independence from the Soviet Union.
Netherlands	B	1981, 1982, 1986, 1989, 1994, 1998, 2002, 2003, 2006, 2010, 2012, 2017	150	–	
Norway	U	1981, 1985, 1989, 1993, 1997, 2001, 2005, 2009, 2013, 2017	169 (from 2005)<br>165 (1989-2001)<br>157 (1985)<br>155 (1981)	–	
Serbia	U	2007, 2008, 2012, 2014, 2016, 2020	250	2006	Independence declared from the Union of Serbia and Montenegro.
Slovakia	U	1992, 1994, 1998, 2002, 2006, 2010, 2012, 2016, 2020	150	1993	Independence from Czechoslovakia.
Slovenia	B	1992, 1996, 2000, 2004, 2008, 2011, 2014, 2018	90	1991	Independence from SFR Yugoslavia.
Spain	B	1989, 1993, 1996, 2000, 2004, 2008, 2011, 2015, 2016, Apr. 2019, Nov. 2019	350	–	
Sweden	U	1982, 1985, 1988, 1991, 1994, 1998, 2002, 2006, 2010, 2014, 2018	349	–	
United Kingdom	B	1983, 1987, 1992, 1997, 2001, 2005, 2010, 2015, 2017, 2019	649 +1 (speaker) (from 2010)<br>645 +1 (2005)<br>658 +1 (2001, 1997)<br>651 (1992)<br>650 (1987, 1983)	–	

(a) M = minority (8), D = diaspora seats (3 to 6).
(b) O = overhangs (since 2005), L = leveling seats (since 2013).
(c) The elections dated 2014 and 2018 have not been considered since the new Constitution of Hungary (2012) changed considerably the number of MPs (hence the frustration is not comparable).
(d) A = seats for Macedonians living abroad (in case of sufficient voter turnout).
(e) The April 2009 election is not considered since no president was elected and new elections had to be held in July 2009.

Supplementary Table 1. List of countries considered in this work. For each country, we list: the structure of the Parliament (B = Bicameral or U = Unicameral), the election dates considered in this study, the number of seats in the Parliament (in case of changes, the year is specified), and the significant events which determined the starting point in our analysis.

**Scenario**	**Correlation between $\lambda_1(\mathcal{L})$ and frustration**	**Overlap (average) between S_{best} and sign $(v_1(\mathcal{L}))$**
**I**	0.956	88.31 %
**II**	0.939	98.12 %
**III**	0.904	97.69 %

Supplementary Table 2. Frustration vs smallest eigenvalue of the normalized signed Laplacian for scenario **I**, **II**, **III**. Left column: average correlation between the frustration of the parliamentary networks ζ and the smallest eigenvalue of the normalized signed Laplacian $\lambda_1(\mathcal{L})$. Right column: average overlap between S_{best} and the signature of $v_1(\mathcal{L})$, the eigenvector associated to $\lambda_1(\mathcal{L})$, as defined in equation (S14).

A

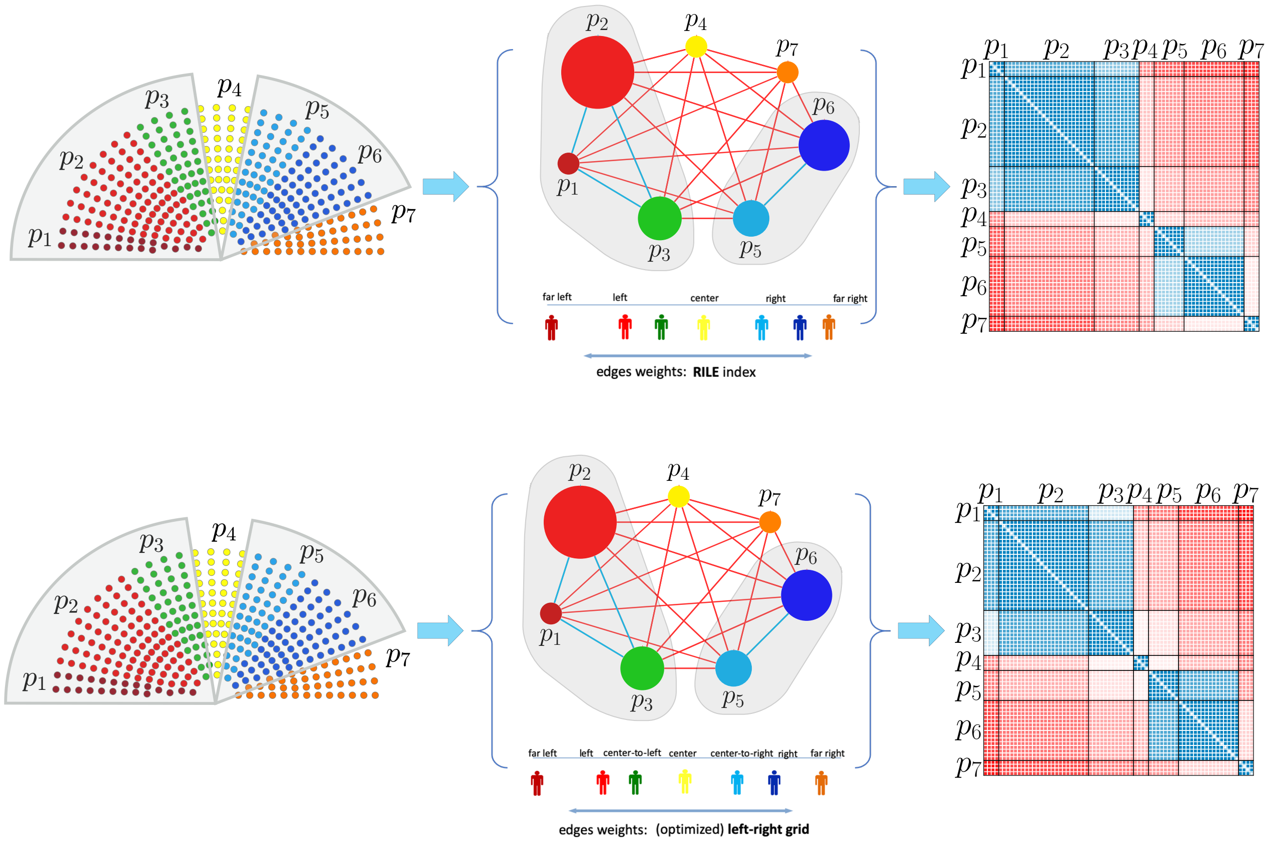

B

Left-right Grid	Political Positions
far-left	$q_{\mathrm{FL}} = -q_{\mathrm{FR}} = -0.5$
left to far-left	$q_{\mathrm{LFL}} = -q_{\mathrm{RFR}}$
left	$q_{\mathrm{L}} = -q_{\mathrm{R}}$
centre-left to left	$q_{\mathrm{CLL}} = -q_{\mathrm{CRR}}$
centre-left	$q_{\mathrm{CL}} = -q_{\mathrm{CR}}$
centre to centre-left	$q_{\mathrm{CCL}} = -q_{\mathrm{CCR}}$
centre	$q_{\mathrm{C}} = 0$
centre to centre-right	q_{CCR}
centre-right	q_{CR}
centre-right to right	q_{CRR}
right	q_{R}
right to far-right	q_{RFR}
far-right	$q_{\mathrm{FR}} = +0.5$
big tent	d_{BT}

C

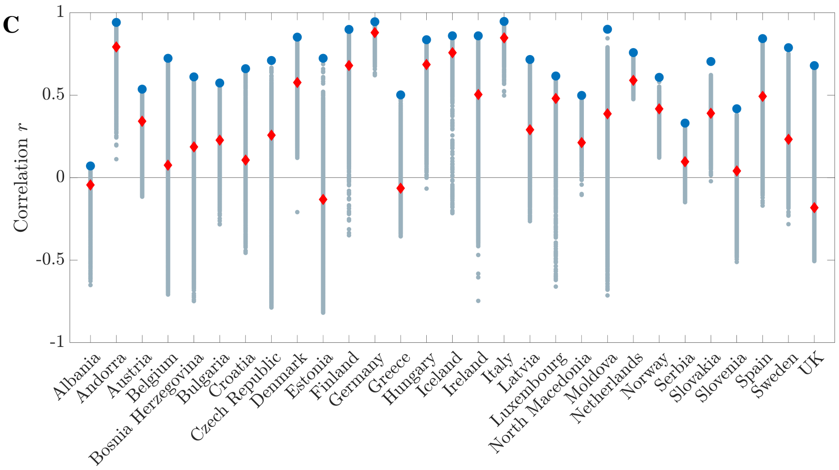

Supplementary Figure 1. (**A**): Constructing a parliamentary network and the corresponding adjacency matrix for scenario **II** (top) and **III** (bottom). Scenario **I** is shown in Fig. 1B of the paper. (**B**): "Political positions" in the left-right political spectrum. (**C**): Correlation r for scenario **III**. 10000 sets of values for the left-right political positions of the parties were randomly selected on the preassigned left-right grid, as explained in Section S1.1. Here the corresponding correlation values for each set are shown (gray circles), together with the overall country mean value (red diamond) and maximum value (blue circle). For each country, the optimal choice for the weights corresponds to the one giving best correlation index (blue circle).

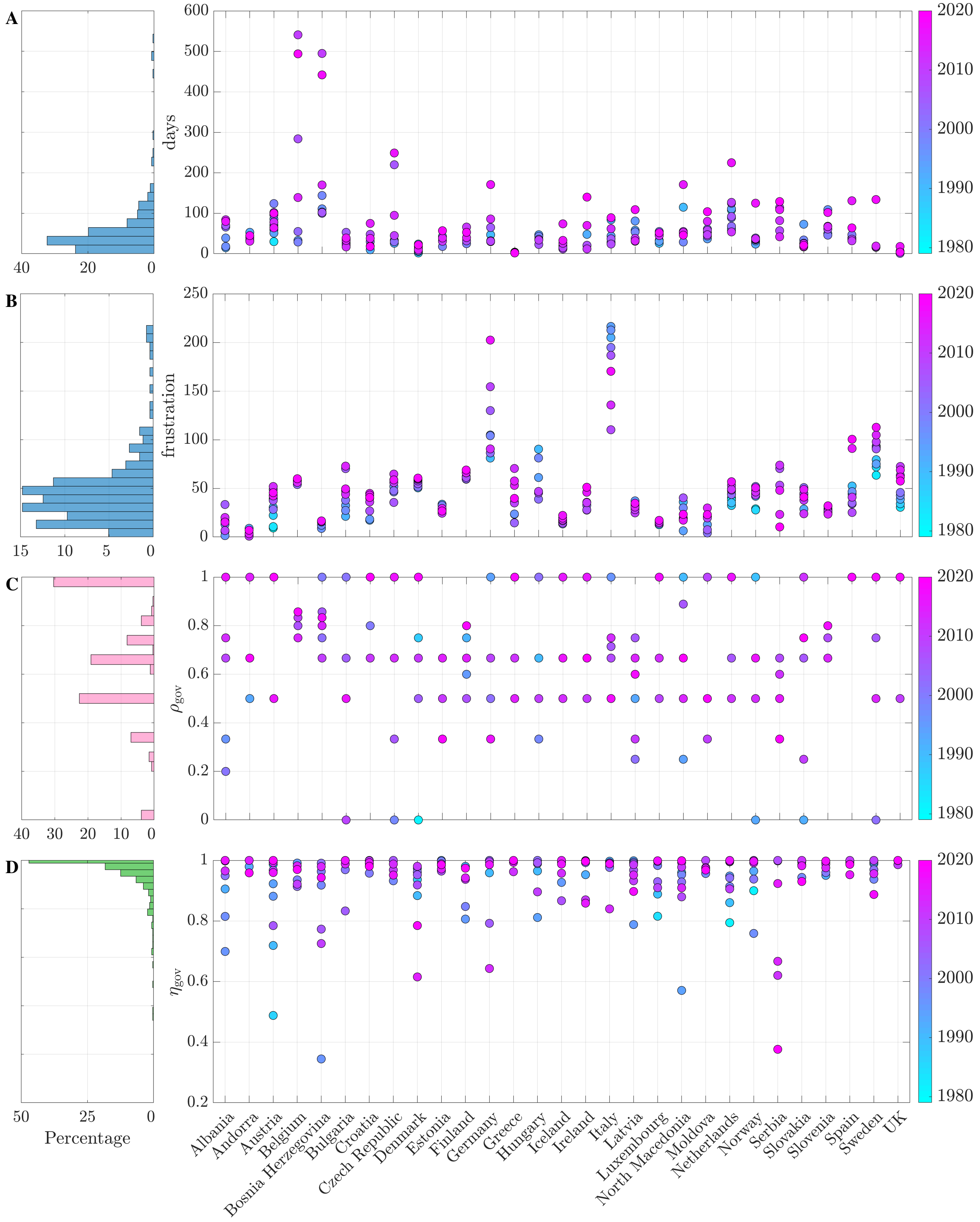

Supplementary Figure 2. (**A**): Number of days of government coalition negotiations for each country and election. (**B**): Party-wise frustration for each country and election in scenario **I**. (**C**) ρ_{gov}, summary for each country and election. (**D**) η_{gov}, summary for each country and election. Left panels in (**C**), and (**D**): corresponding histograms. A colormap is used to differentiate data from different years.

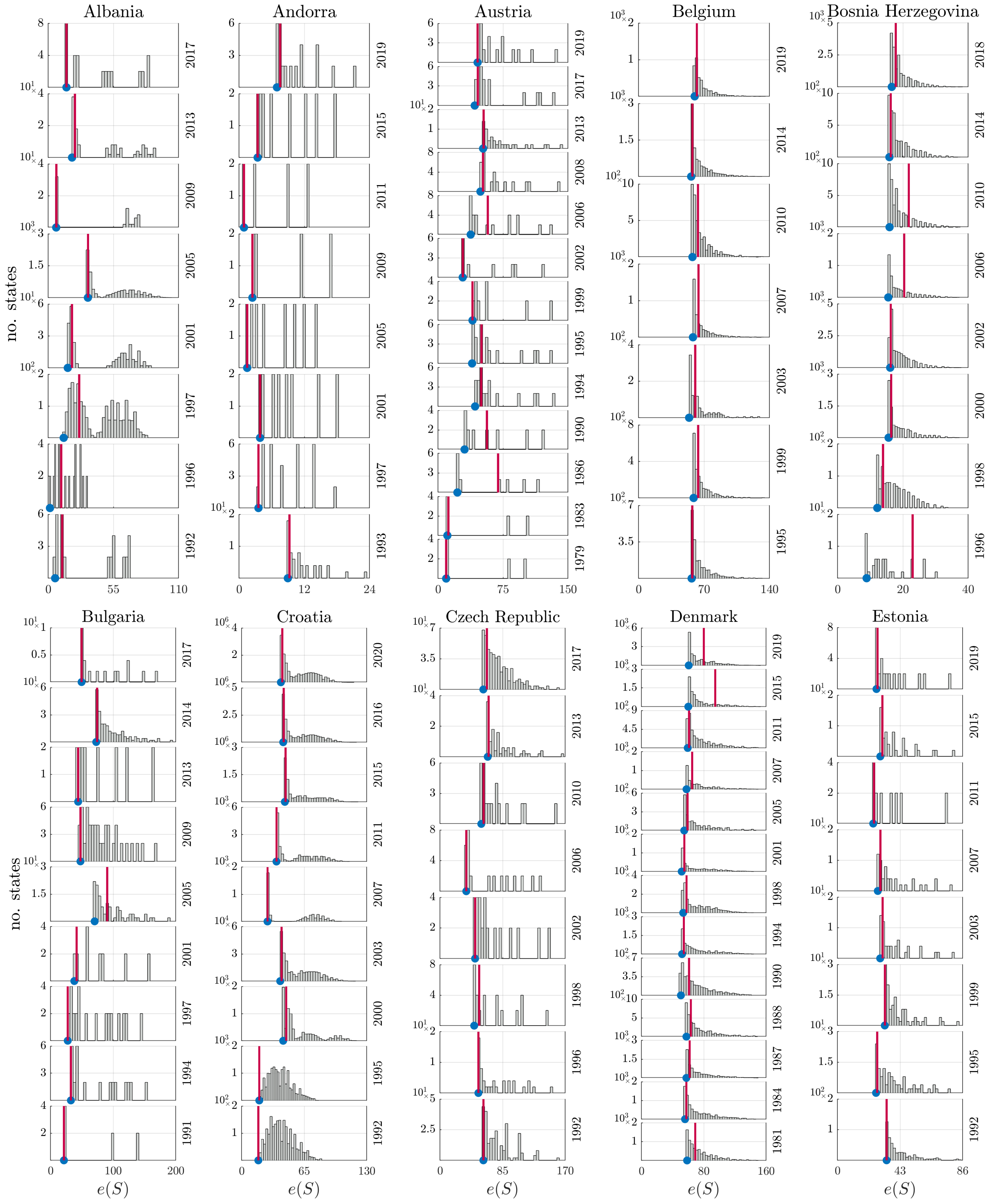

Supplementary Figure 3. Continued on the next page.

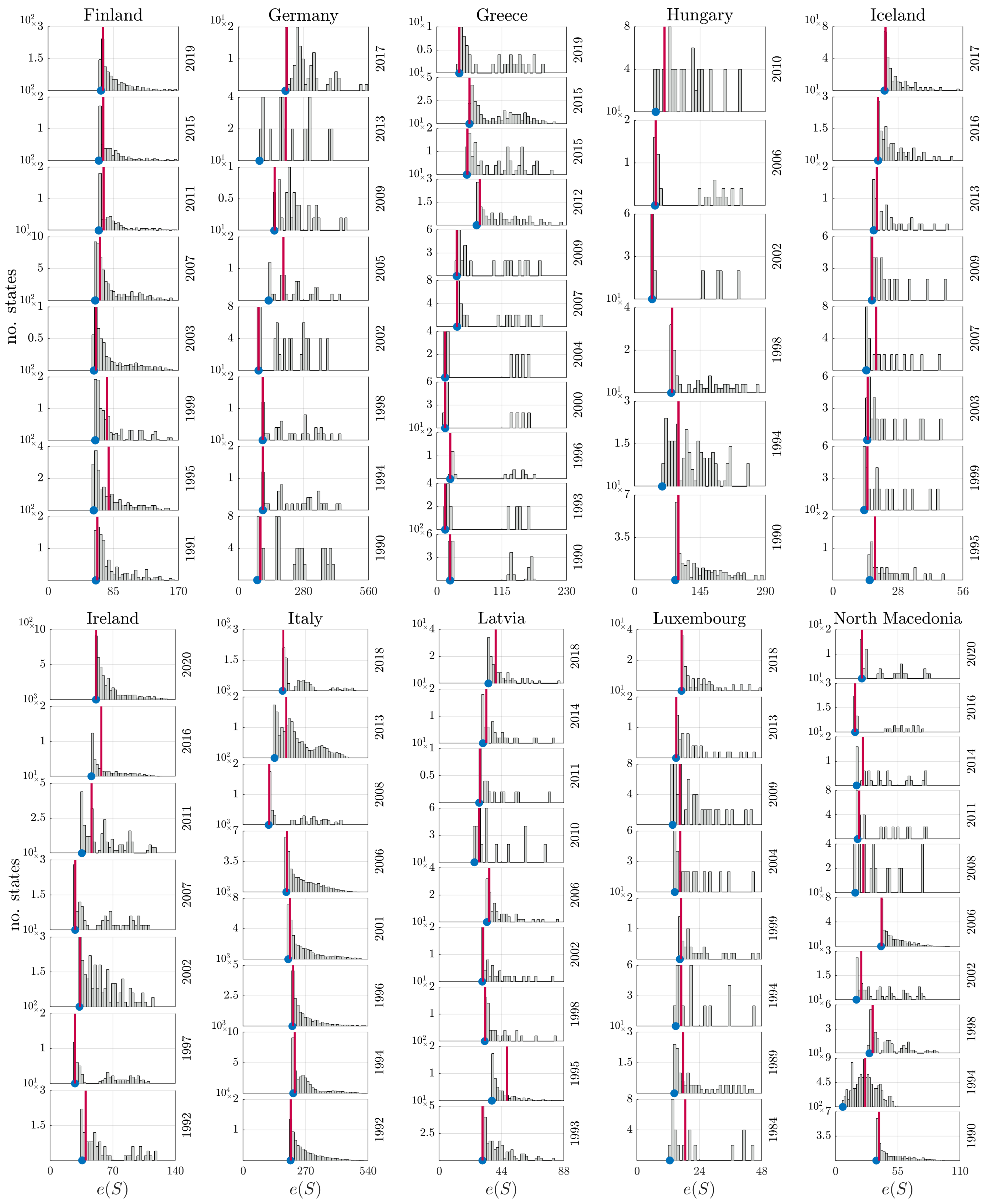

Supplementary Figure 3. Continued on the next page.

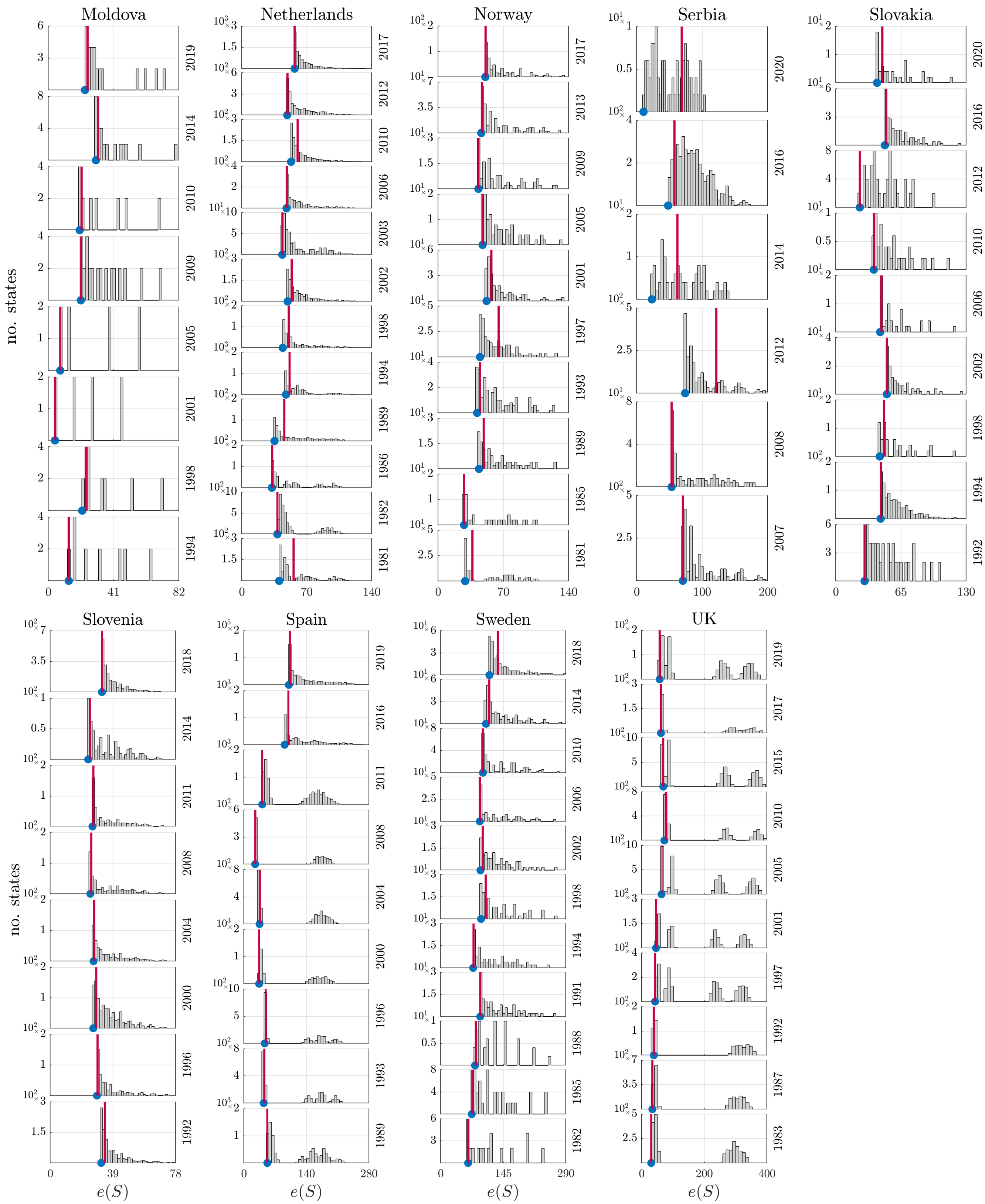

Supplementary Figure 3. Energy landscape for scenario **I** and for each country, i.e., values of the energy $e(S)$ (see (S6)) as $S = \mathrm{diag}\{s_1 I_{c_1}, \ldots, s_{n_\mathrm{p}} I_{c_{n_\mathrm{p}}}\}$, $s_i = \pm 1$, is varied: the value of the energy functional corresponding to the government, $e(S_\mathrm{gov})$, is highlighted with the red line, while the minimum of the energy functionals, $e(S_\mathrm{best}) = \zeta$, is indicated with the blue dot.

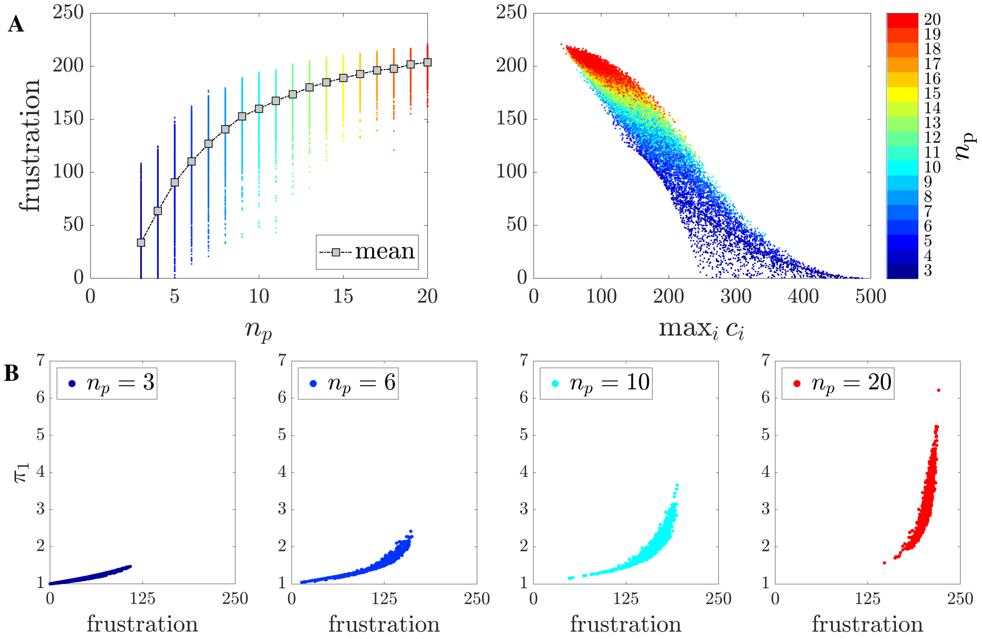

Supplementary Figure 4. Numerical example of computation of the frustration as the number of parties and of seats per party varies. (**A**): Party-wise frustration ζ of the network $\mathcal{G}$ vs number of parties (n_p) and vs maximum number of MPs per party ($\max_i c_i$), as the number of parties is changed and the number of seats per party is varied randomly. The artificial networks we consider here are "all-against-all" networks with $n_\mathrm{p} \in \{3, \dots, 20\}$ parties and size $n = 500$. (**B**): Behavior of π_1 as a function of the frustration ζ, as the number of seats per party changes randomly and $n_\mathrm{p} \in \{3, 6, 10, 20\}$.

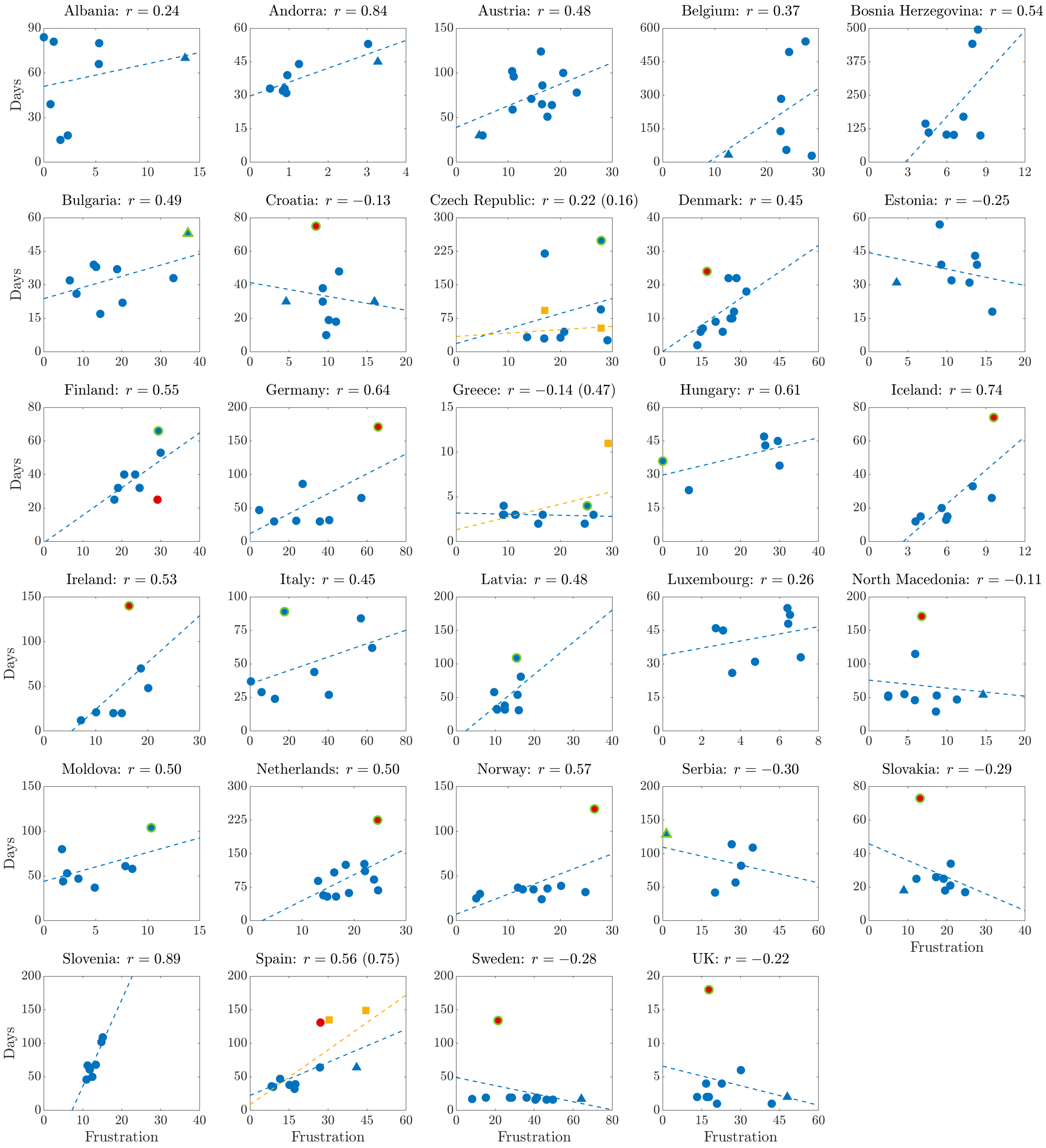

Supplementary Figure 5. Scenario **II**. Frustration of the parliamentary networks v.s. duration of the government negotiation talks (days) and corresponding linear regression line, for all countries of Supplementary Table 1. The value of correlation (r) for each country is reported in the plot heading. Legend: blue circles represent points that are neither outliers, nor high leverage nor influential. A red symbol indicates an outlier, a triangle a high leverage point and a symbol with green outline an influential point. Residual analysis, leverage statistic and delete-1 statistics are used to identify outliers, high leverage and influential points, respectively.
Yellow square data points indicate elections corresponding to failure of government negotiations resulting in votes of no-confidence (Czech Republic in 2006 and 2017) and new elections (Spain in December 2015 and April 2019, Greece in May 2012), see Supplementary Fig. 7A. Blue regression lines consider only the successful government formations. Including also the failure points we obtain the yellow regression lines.

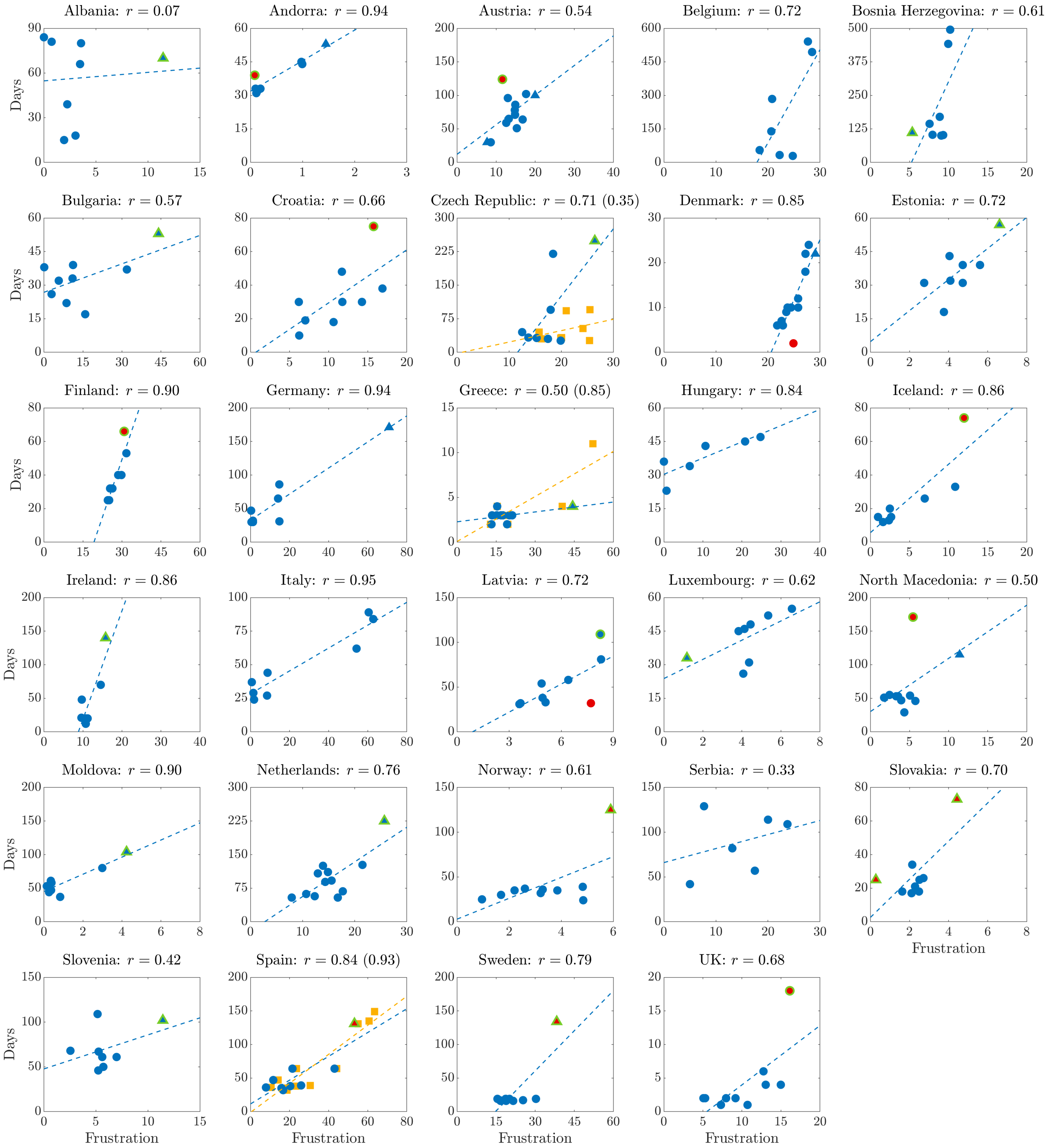

Supplementary Figure 6. Scenario **III**. Frustration of the parliamentary networks v.s. duration of the government negotiation talks (days) and corresponding linear regression line, for all countries of Supplementary Table 1. The value of correlation (r) for each country is reported in the plot heading. Legend: blue circles represent points that are neither outliers, nor high leverage nor influential. A red symbol indicates an outlier, a triangle a high leverage point and a symbol with green outline an influential point. Residual analysis, leverage statistic and delete-1 statistics are used to identify outliers, high leverage and influential points, respectively.

Yellow square data points include also elections corresponding to failure of government negotiations resulting in votes of no-confidence (Czech Republic in 2006 and 2017) and new elections (Spain in December 2015 and April 2019, Greece in May 2012), see Supplementary Fig. 7A. In these 3 countries all edge weights have been recomputed (hence frustration values are different with respect to the blue data points). Blue regression lines consider only the successful government formations. Including also the failure points we obtain the yellow regression lines.

A

Country	Date			No. Days
	election	failure	sworn-in	
Czech Republic	2006-06-03	2006-09-04	2007-01-09	93 (failure), 220 (success)
	2017-10-21	2017-12-13	2018-06-27	53 (failure), 249 (success)
Greece	2012-05-06	2012-05-17	–	11 (failure)
	2012-06-17	–	2012-06-21	4 (success)
Spain	2015-12-20	2016-05-03	–	135 (failure)
	2016-06-26	–	2016-11-04	131 (success)
	2019-04-28	2019-09-24	–	149 (failure)
	2019-11-10	–	2020-01-13	64 (success)

B

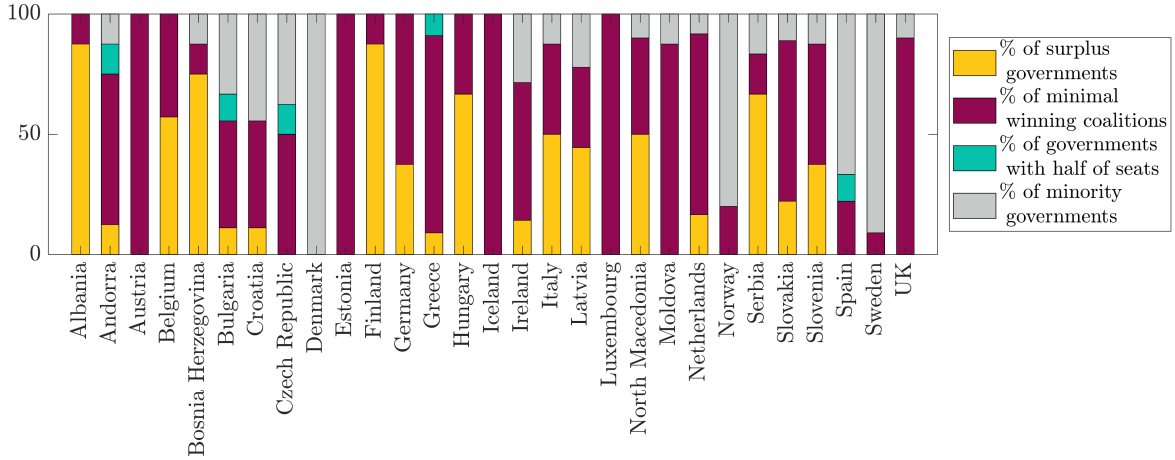

Supplementary Figure 7. (**A**): Legislative elections related to failure of government negotiation talks in Czech Republic, Greece and Spain. (**B**): Type of government coalition formed after the election (minority, minority but yielding exactly half of the seats in the parliament, minimal winning, surplus), for each country of Supplementary Table 1. Observe that to be classified as "majority" a cabinet coalition needs to hold (strictly) more than half of the seats in the parliament.

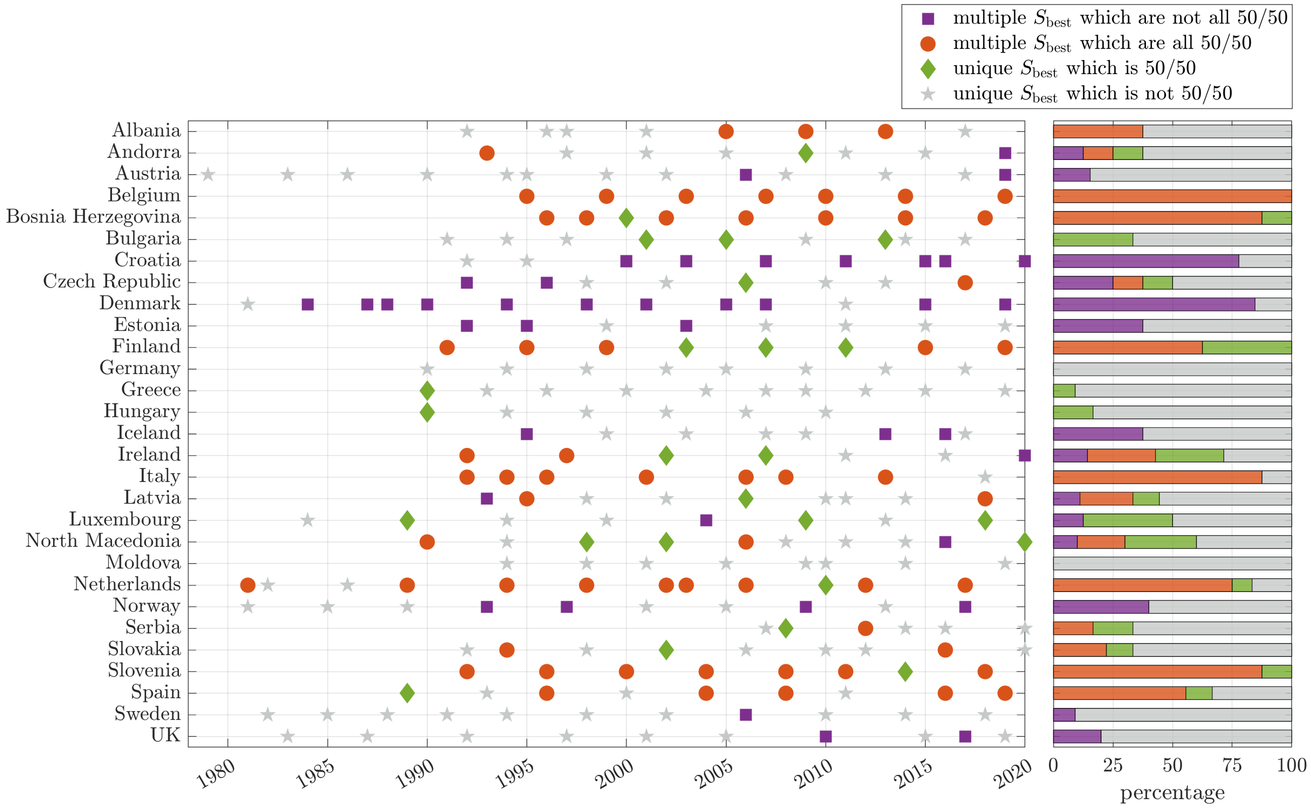

Supplementary Figure 8. Scenario **I**. Elections for which the ground state (S_{best}) is "degenerate" (purple, orange, green) and not "degenerate" (light gray). Left panel: cases in which the ground state (S_{best}) is "degenerate" for each country and election; Right panel: percentage of elections for which the ground state is "degenerate", for each country. Legend: gray stars are used to represent cases where the ground state is not "degenerate". We consider three different "degenerate" cases: (1) multiple signature matrices S_{best} give the same value of frustration but there exists a S_{best} whose corresponding group of parties holds a majority of seats in the parliament (purple squares); (2) multiple signature matrices S_{best} give the same value of frustration and all corresponding party groups hold exactly half of the seats (50/50) in the parliament (orange circles); (3) unique signature matrix S_{best} whose corresponding party group holds exactly half of the seats (50/50) in the parliament (green diamond).

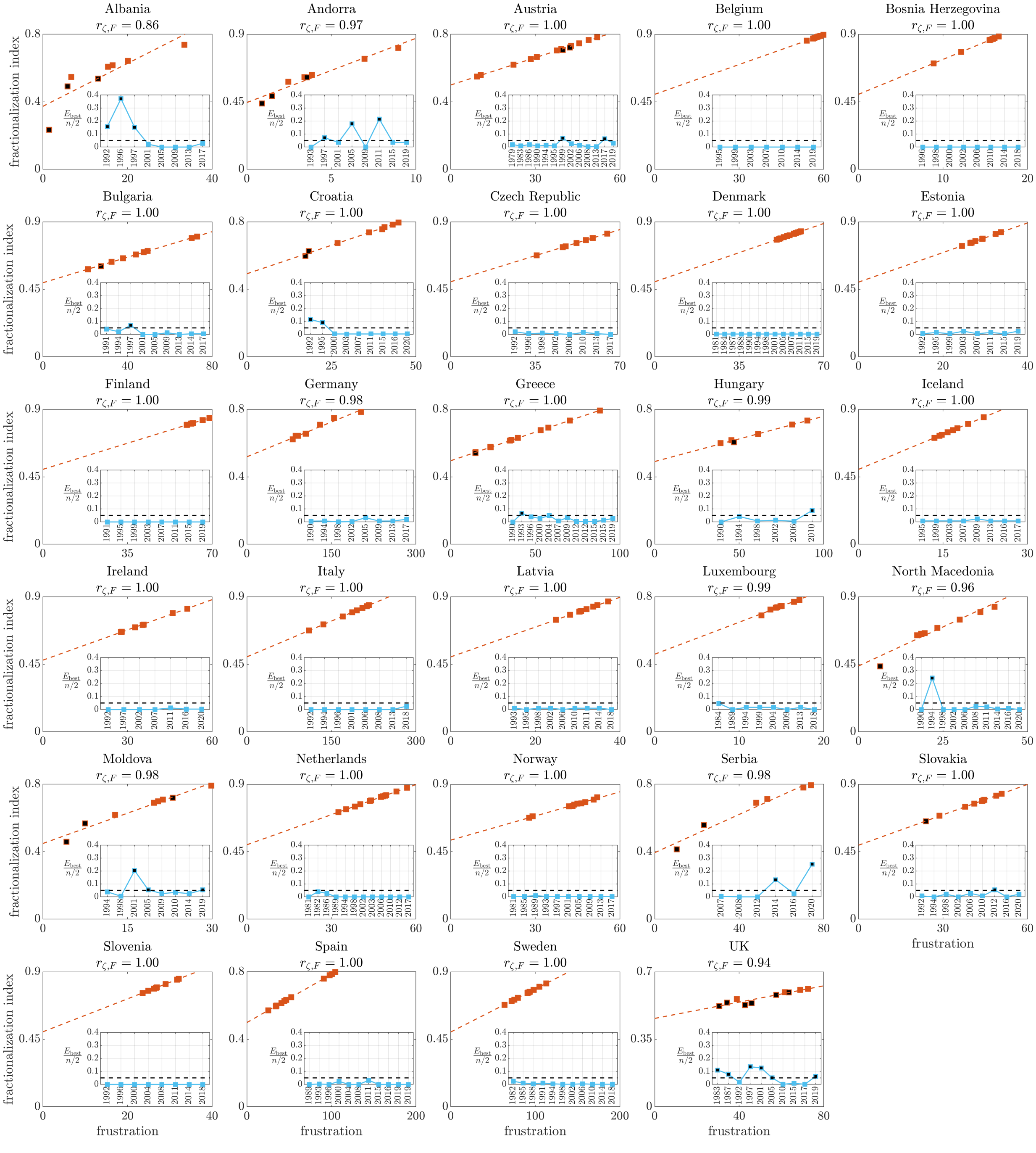

Supplementary Figure 9. Scenario **I**. Fractionalization index vs frustration of the parliamentary networks and corresponding linear regression line, for all countries of Supplementary Table 1. The value of correlation ($r_{\zeta,F}$) for each country is reported in the plot heading. Inset: distance of the minimum winning coalition S_{best} to 50% (of the total number of seats), calculated as $\frac{E_{\text{best}}}{n/2}$ where E_{best} is the number of seats in excess of S_{best} (see Section S2.4). In all panels black squares indicate minimum winning coalitions S_{best} whose distance from 50% is greater than 5%.

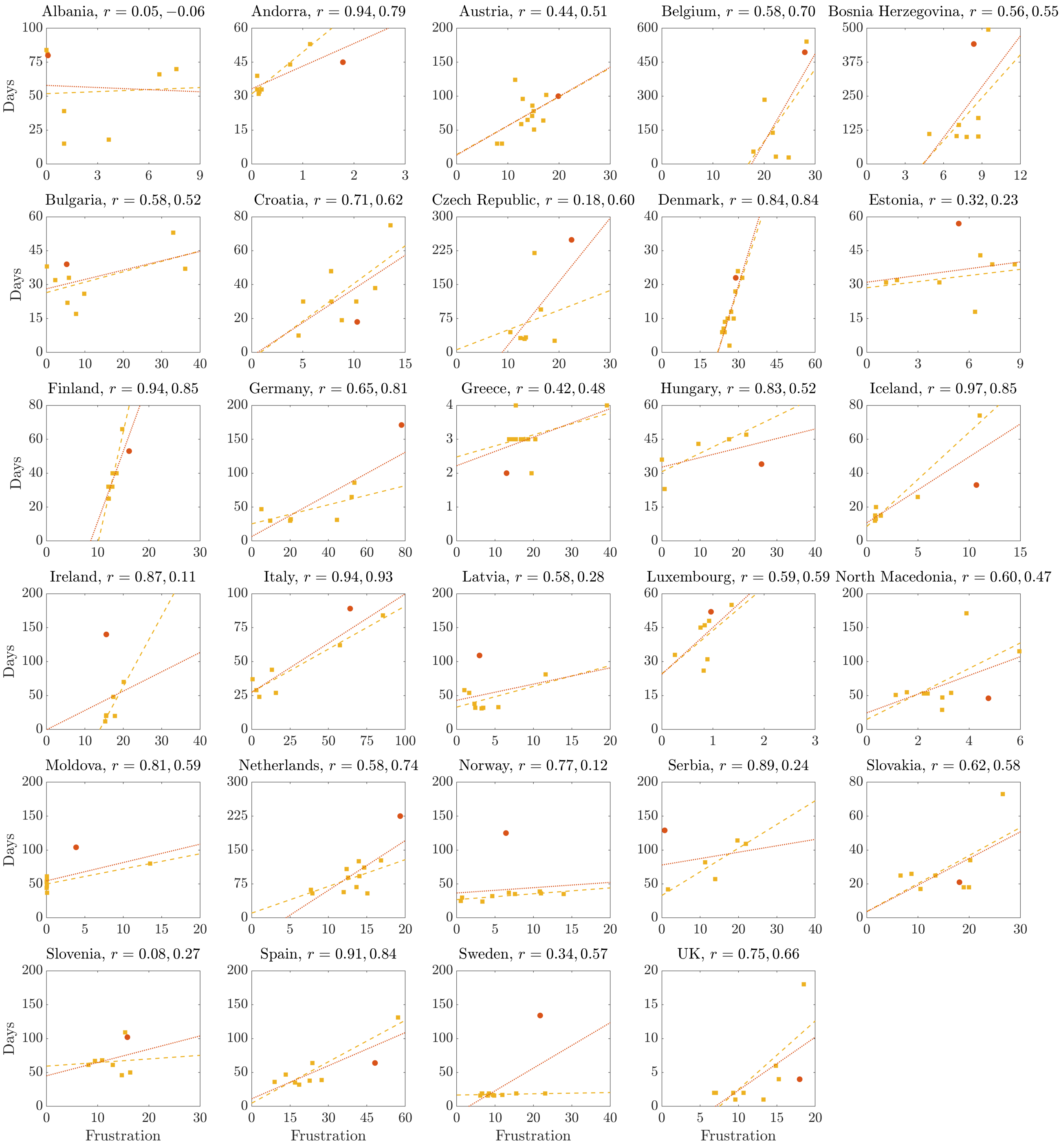

Supplementary Figure 10. Leave-the-last-one-out analysis and linear regression plots between the duration of the government negotiation talks (days) and the frustration of the parliamentary networks in scenario **III**, for all countries. The last election (red circle) is used for the validation set while the remaining elections (yellow squares) are used to calculate the optimal choice of political positions in the left-right grid (see Fig. 4 in the main paper). A yellow dashed line represents the regression calculated on the first $N-1$ elections, while a red dotted line the regression calculated on all elections. The corresponding values of correlation r are reported in the plot heading.
